# Deregulation of Exo70 Facilitates Innate and Acquired Cisplatin Resistance in Epithelial Ovarian Cancer by Promoting Cisplatin Efflux

**DOI:** 10.3390/cancers13143467

**Published:** 2021-07-11

**Authors:** Yujie Zhao, Xiaoting Hong, Xiong Chen, Chun Hu, Weihong Lu, Baoying Xie, Linhai Zhong, Wenqing Zhang, Hanwei Cao, Binbin Chen, Qian Liu, Yanyan Zhan, Li Xiao, Tianhui Hu

**Affiliations:** 1 Department of Oncology, Zhongshan Hospital Affiliated to Xiamen University, Xiamen 361004, China; 24520170155607@stu.xmu.edu.cn; 2 Cancer Research Center, School of Medicine, Xiamen University, Xiamen 361102, China; xthong@xmu.edu.cn (X.H.); 24520170155572@stu.xmu.edu.cn (X.C.); xbying@xmu.edu.cn (B.X.); 24520180155713@stu.xmu.edu.cn (L.Z.); wqzhang@xmu.edu.cn (W.Z.); 24520181154539@stu.xmu.edu.cn (H.C.); cbb@xmu.edu.cn (B.C.); yyzhan@xmu.edu.cn (Y.Z.); 3 Department of Oncology, Xiamen Humanity Hospital, Fujian Medical University, Xiamen 361009, China; kejiao@haxm.com; 4 Department of Obstetrics and Gynecology, Xiamen Branch, Zhongshan Hospital, Fudan University, Xiamen 361015, China; lu.weihong@zsxmhospital.com; 5 Key Laboratory of the Education Ministry for the Prevention and Treatment of Cardiovascular and Cerebrovascular Diseases, Ganan Medical University, Ganzhou 341000, China; xiemingfeng@gmu.edu.cn

**Keywords:** Exo70, cisplatin resistance, epithelial ovarian cancer, autophagy

## Abstract

**Simple Summary:**

Innate and acquired platinum resistance are the leading causes of epithelial ovarian cancer (EOC) mortality. However, the mechanisms remain elusive. Here we found that Exo70, a key subunit of the exocyst, is upregulated in EOC and promotes cisplatin efflux to facilitate innate resistance. More interestingly, cisplatin could downregulate Exo70 to sustain cell sensitivity. However, this function was hampered during prolonged cisplatin treatment, which in turn stabilized Exo70 to facilitate the acquired cisplatin resistance of EOC cells. Our study potentiates Exo70 as a promising target to overcome cisplatin resistance in EOC.

**Abstract:**

Whilst researches elucidating a diversity of intracellular mechanisms, platinum-resistant epithelial ovarian cancer (EOC) remains a major challenge in the treatment of ovarian cancer. Here we report that Exo70, a key subunit of the exocyst complex, contributes to both innate and acquired cisplatin resistance of EOC. Upregulation of Exo70 is observed in EOC tissues and is related to platinum resistance and progression-free survival of EOC patients. Exo70 suppressed the cisplatin sensitivity of EOC cells through promoting exocytosis-mediated efflux of cisplatin. Moreover, cisplatin-induced autophagy-lysosomal degradation of Exo70 protein by modulating phosphorylation of AMPK and mTOR, thereby reducing the cellular resistance. However, the function was hampered during prolonged cisplatin treatment, which in turn stabilized Exo70 to facilitate the acquired cisplatin resistance of EOC cells. Knockdown of Exo70, or inhibiting exocytosis by Exo70 inhibitor Endosidin2, reversed the cisplatin resistance of EOC cells both in vitro and in vivo. Our results suggest that Exo70 overexpression and excessive stability contribute to innate and acquired cisplatin resistance through the increase in cisplatin efflux, and targeting Exo70 might be an approach to overcome cisplatin resistance in EOC treatment.

## 1. Introduction

Ovarian cancer is a molecularly heterogeneous disease with the highest mortality among gynecologic malignancies. About 85–90% of ovarian cancer cases are epithelial ovarian cancer (EOC), histologically classified into four major subtypes: serous, mucinous, clear cell, and endometrioid carcinomas [1]. Of these subtypes, high-grade serous ovarian cancer (HGSC) is the most common one and is often diagnosed at an advanced stage with poor survival outcomes [2]. Standard front-line treatments for EOC are cytoreductive surgery and platinum-based chemotherapy [3]. Among those platinum-based drugs, cisplatin is the first approved by the FDA for cancer chemotherapy and is currently used as the first-line drug for EOC treatment [4]. Although the majority of patients with EOC respond to first-line chemotherapy, 80% of the patients relapse and develop platinum resistance, with a median progression-free survival of 12–18 months [3]. New therapeutic methods have recently been introduced into clinical trials to treat recurrent EOC, including VEGF inhibitors, poly-ADP ribose polymerase inhibitors (PARPi), antibody-drug-conjugates (Mirvetuximab soravtansine), and immunotherapy. However, these approaches’ therapeutic effects still greatly depend on the patients’ responses to platinum [5,6,7,8,9]. Moreover, second-line treatment for patients with relapse is also stratified according to the platinum-free interval (PFI); patients with platinum resistance usually have fewer second-line treatment options and worse prognoses [5,10,11]. Thus, platinum resistance is one of the major obstacles to be overcome in EOC treatment. Detecting the mechanisms of platinum resistance is the main challenge owing to the heterogeneity of tumors and the evolvement of resistance over time. Predictive factors are also in great need to guide treatment decisions.

Platinum-based chemotherapy works through binding nuclear DNA and consequently activates cellular DNA damage response (DDR) pathways to induce cell cycle arrest and apoptosis [12,13]. Platinum resistance is related to a variety of intercellular mechanisms and molecules, including the decrease in drug uptake (by copper transport 1 (CTR1) and organic cation transporters (OCT)), the increase in drug efflux (by ATPase copper transporting alpha (ATP7A), ATPase copper transporting beta (ATP7B)), and multidrug resistance protein (MRP)), the intracellular deactivation of the drug (by glutathione (GSH) and metallothionein), the impairment of DNA repair, the defects in apoptosis response, and epithelial-mesenchymal transition [14]. Among them, upregulation of drug efflux pumps (such as ATP7A, ATP7B, and MRP) leading to insufficient intracellular chemotherapy concentration is an important reason for the platinum resistance of EOC [15,16,17]. Cisplatin can also be trapped into non-secreted vesicles around Golgi by ATP7A/ATP7B to decrease its effective concentration for DNA binding [18]. Lysosomal exocytosis is another mechanism to facilitate the clearance of platinum highly accumulated in lysosomes [19]; however, the molecules involved in the exocytosis of cisplatin-loaded lysosomes remain unknown.

The exocyst, an octameric complex consisting of Sec3, Sec5, Sec6, Sec8, Sec10, Sec15, Exo70, and Exo84, mediates the docking of secretory vesicles to the plasma membrane during exocytosis [20]. Interaction of its subunit Exo70 with PI(4,5)P2 in the plasma membrane is critical for vesicle tethering [21]. Exo70 is also reported to accelerate polarized lysosome secretion at the immune synapse and promote cell migration and invasion in multiple types of tumors [22,23,24,25,26]. In previous studies, we reported that Exo70 affects the progression of liver cancer and breast cancer [27,28]. However, it remains to be seen whether Exo70 is involved in the tumorigenesis, progression, or platinum resistance of EOC.

In the present study, we reported Exo70 as a potential predictive marker and a therapeutic target for both innate and acquired cisplatin resistance in EOC treatment. Elevated Exo70 expression is clinically related to innate cisplatin resistance of EOC. Exo70 reduced cisplatin sensitivity by promoting exocytosis-mediated cisplatin efflux. Interestingly, cisplatin in turn induced autophagy-lysosome degradation of Exo70 protein to overcome the efflux. However, the degradation of Exo70 was hampered during prolonged cisplatin treatment and thus contributed to acquired resistance. Exo70 inhibitor Endosidin2 (ES2) was further proved to overcome acquired cisplatin resistance of EOC cells in vitro and in vivo, and would further be developed as a lead compound to improve the clinical chemotherapeutic efficacy of cisplatin in EOC. Our study potentiates Exo70 as a promising target to overcome cisplatin resistance in EOC.

## 2. Materials and Methods

### 2.1. Cell Culture

Human embryonic kidney cell line HEK293T and human ovarian cancer cell lines OVCAR3 and SK-OV-3 were purchased from the Institute of Cell Biology, Chinese Academy of Sciences (Shanghai, China). Human ovarian cancer cell line A2780 was purchased from ATCC (Manassas, VA, USA). All cell lines were identified by Genetic Testing Biotechnology Corporation (Suzhou, China) using short tandem repeat (STR) markers. STR profiling reports of cell lines are shown in Figure S8. The cisplatin-resistant cell line A2780CR was established by culturing in indicated doses of cisplatin. HEK293T, A2780, and A2780CR cells were cultured in Dulbecco’s modified Eagle medium (Gibco, Grand Island, NY, USA) supplemented with 10% Fetal Bovine Serum (FBS) (Gibco, Grand Island, NY, USA). OVCAR3 were cultured in RPMI-1640 medium (Gibco, Grand Island, NY, USA) supplemented with 20% FBS.SK-OV-3 cells were cultured in McCoy’s 5A medium (Gibco, Grand Island, NY, USA) supplemented with 10% FBS. All the medium was supplemented with 100 U of penicillin and 100 μg/mL of streptomycin (Life Technologies, Carlsbad, CA, USA). All cells were incubated at 37 ℃ in a 5% CO2 incubator.

### 2.2. Antibodies and Reagents

The information on antibodies was listed in Table S1. Cycloheximide (Cat. #239763-M) and Rapamycin (Cat. #R0395) were purchased from Sigma-Aldrich. Chloroquine (Cat. #HY-17589A), Bafilomycin A1 (Cat. #HY-100558), and MG132 (Cat. #HY-13259) were purchased from MCE (Monmouth Junction, NJ, USA). Endosidin2 (ES2) (Cat. #21888) was purchased from Cayman Chemical (Ann Arbor, MI, USA).

### 2.3. Vector Construction and Generation of Stable Cell Lines

Human Exo70 was Flag, mCherry, or GFP tagged by PCR and subcloned into pCMV10 (RRID: Addgene_51888) or pLV (RRID: Addgene_85140) vector. Ubiquitin (Ub) was Myc-tagged by PCR and subcloned into pcDNA3.0 (RRID: Addgene_12452) vector. shRNA were synthesized and cloned into pLKO.1 (RRID: Addgene_52920) vector. The sequences of shRNAs were listed in Table S2. Lentiviruses were generated by transfecting 293T cells with pLKO.1 vector encoding shRNA or pLV vector encoding Exo70, together with package vectors pMDL, pVSVG, and pRSV-Rev (RRID: Addgene_12253). Viruses were collected and infected cells. Stable cell lines were established by selection of 2 μg/mL puromycin.

### 2.4. Cell Viability Assay and Colony Formation Assay

Cell counting kit-8 (CCK-8) assay (Beyotime, Shanghai, China) was used to determine cell viability and drug sensitivity according to the manufacturer’s instructions. Briefly, cells were seeded in a 96-well microplate at a density of 3000/well. After 12 h, cells were treated and cultured as indicated. CCK-8 solution was added and the plate was incubated at 37 ℃ for 2 h. The viable cells were counted by the absorbance at a wavelength of 450 nm using a microplate reader. The inhibition rate of cisplatin was calculated as follows: (1 − [absorbance of the cisplatintreated group/absorbance of the untreated group]) × 100.

For the colony-forming assay, 500 cells were seeded in 6-well plates and treated with cisplatin with indicated concentrations. Then, 1 h after treatment, the cells were washed with PBS and cultured for 10 days. Colonies were then fixed with 4% paraformaldehyde and stained with 1% crystal violet. Clones containing over 50 cells were counted. Each result was performed in triplicate.

### 2.5. Apoptosis Assay

Cells were treated with cisplatin. After 24 h, cells were collected, and apoptotic cells were assessed by TUNEL Apoptosis Detection kit (Vazyme, Cat. #A113) or PE-Annexin V/7-AAD Apoptosis Detection kit (BD Biosciences, Cat. #559763) according to the manufacture’s instruction.

### 2.6. Western Blot and Immunoprecipitation

Cells were lysed in RIPA buffer (150 mM NaCl, 50 mM Tris pH7.4, 1% NP-40, 0.5% sodium deoxycholate and 0.1% SDS, 1 mM PMSF and protease inhibitors). The protein concentration of cell lysates was determined by BCA kit (Thermo Scientific, Rockford, IL, USA). An equal amount of protein was analyzed by SDS-PAGE, and then transferred to nitrocellulose membrane. The membrane was blocked in TBS-T containing 5% nonfat milk at room temperature for 1 h and then incubated with antibodies. Immunopositive bands were detected by Enhanced Chemiluminescence (ECL) system (Bio-Rad, Hercules, CA, USA). For immunoprecipitation, cell lysates were incubated with the antibody in the presence of A/G-agarose beads overnight. The immunoprecipitation beads were then washed four times with lysis buffer, followed by western blotting analysis. The uncropped Western blots have been shown in Figures S6 and S7.

### 2.7. Real-Time PCR

Total RNA was isolated using Trizol reagent (TaKaRa, Dalian, China). cDNA was synthesized using a Primescript™ RT reagent kit (TaKaRa, Dalian, China). Real-time PCR was performed using the SYBR Green I fluorescent dye (TaKaRa, Dalian, China). The primer pairs were listed in Table S3.

### 2.8. FITC-Cisplatin Uptake

FITC-cisplatin was constructed according to previous literature [29]. Cells were incubated with FITC-cisplatin for 12 h. Cells were then washed with PBS and visualized in Zeiss LSM 880 Laser Scanning Confocal Microscope.

### 2.9. Dot-Blot

Pt-DNA adduct detection was carried out by dot-blot according to Wang et al. [13]. Cells were treated with cisplatin. After 24 h, the genomic DNA of cells was isolated using phenol-chloroform-isoamyl alcohol. Equal amounts of DNA were spotted onto nitrocellulose membrane and baked at 80 ℃ for 2 h. The membrane was then incubated with anti-CDDP adducts antibody (clone ICR4, EMD Millipore), then washed with TBST, and incubated with an HRP-conjugated secondary antibody. Chemiluminescent signals were detected by Enhanced Chemiluminescence (ECL) system (Bio-Rad, Hercules, CA, USA).

### 2.10. Platinum Accumulation and Efflux Assay

Cells were treated with cisplatin for 3 h and collected by trypsinization. The pellets were washed with D-Hanks and digested in 70% nitric acid overnight at room temperature. Platinum concentration in these pellets was determined by inductively coupled plasma mass spectrometry (ICP-MS, Agilent 7500CE) and considered as the intracellular accumulation of cisplatin (Ptc). To detect the efflux percentage, an equal number of cells were treated with cisplatin for 3 h. The medium was replaced with fresh medium and cultured for another 12 h. The supernatant was collected, digested, and measured by ICP-MS to determine the platinum concentration (Pts). Indium was added as an internal standard. The ratio of Pts and Ptc was calculated as the efflux percentage of platinum.

### 2.11. Isolation of Extracellular Vesicles from Cultured Cells

Extracellular vesicles were isolated as described by Jeppesen DK et al. [30]. Cells were cultured in the medium supplemented with exosome-depleted fetal bovine serum for 12 h and then treated with cisplatin. After 3 h treatment, cells were washed with D-Hanks buffer three times. Fresh medium was added and cultured for another 12 h. The cell-conditioned medium was collected and sequentially centrifuged at 400× *g* 10 min and 2000× *g* for 20 min at 4 ℃ to remove cells and debris/apoptotic bodies, respectively. To collect large extracellular vesicles (LEVs), the supernatant was centrifuged at 15,000× *g* for 40 min, and the pellet was washed by resuspending in phosphate-buffered saline followed by centrifugation at 15,000× *g* for 40 min. This pellet was collected as LEVs. To collect small extracellular vesicles (SEVs), the supernatant collected from the first 15,000× *g* step was passed through a 0.22 μm pore PES filter (Millipore) to remove any remaining LEVs, and next subjected to ultracentrifugation at 120,000× *g* for 4 h in an SW32 Ti swinging bucket rotor. The supernatant was collected for analysis and the crude SEVs pellet was washed with PBS in the same condition. During the process of collecting extracellular vesicles, all the samples were subjected to temperatures below 4 ℃.

### 2.12. Animal Studies

All animal experiments were conducted following protocols approved by the Animal Care and Use Committee of Xiamen University. The animal ethics number is XMULAC20170233. Balb/c nude mice aged 6 weeks were subcutaneously inoculated with OVCAR3 cells (1 × 10^6^ in 100 μL RPMI-1640 per mouse) or SK-OV-3 cells (2 × 10^6^ in 100 μL McCoy’s 5A per mouse). After tumors were established, cisplatin (6 mg/kg) was administrated intraperitoneally every three days, and tumor size was measured using calipers. Tumors were collected and processed for IHC and HE after 21 days of treatment. A2780CR cells were intraperitoneally inoculated into 5-week old female nude mice (3 × 10^6^ cells in 100 μL DMEM per mouse). Cisplatin (6 mg/kg) and ES2 (6 mg/kg) were administered intraperitoneally every six days. After 24 days of treatment, mice were euthanized, and the peritoneal metastasic tumors were collected and measured. For in vivo bioluminescent imaging, mice were administrated with 150 mg/kg D-luciferin (Promega, Cat. #E1605) and imaged by Caliper IVIS Lumina II Kinetic system (Caliper Life Sciences, Waltham, MA, USA), and the total photon flux was calculated by Living Image 4.4.

### 2.13. Clinical Tissue Analysis

Immunohistochemical staining for Exo70 was performed on the ovarian cancer tissue microarrays, which included 61 EOC samples (Histologic types: Serous (40), Mucinous (9), Endometrioid (6), and Clear cell (6); Cancer differentiation grades: Grade 1 (8), Grade 2 (29), Grade 3 (23), Grade 4 (1)) and 15 normal ovarian tissue samples, obtained from the tissue bank of Zhongshan Hospital Affiliated to Xiamen University. Ten platinum-sensitive and resistant tissue samples were also collected from Zhongshan Hospital Affiliated to Xiamen University. Characteristics of the samples isolated from ovarian cancer patients were listed in Table S4. The studies were approved by the Medical Ethics Committee of Zhongshan Hospital Affiliated to Xiamen University in accordance with the Helsinki Declaration, and conducted with the informed consent of all patients. The ethical approval number is 20190003.

### 2.14. Immunohistochemistry (IHC) and Hematoxylin-Eosin (HE) Staining

Tumor tissues were isolated, fixed in 10% formalin, embedded in paraffin, and cut into 4 μm paraffin-embedded sections for IHC and HE assay. For IHC, sections were subjected to antigen retrieval in 10 mmol/L sodium citrate buffer (pH 6.0) in a microwave oven. Sections were then incubated with the primary antibody at 4 ℃ overnight and with a biotinylated secondary antibody for 30 min, followed by the avidin–biotin–peroxidase complex for another 10 min. Staining was visualized using 3,3′-diaminobenzidine solution (Maxim, Fuzhou, China). For HE, sections were stained with hematoxylin-eosin, and images were obtained with Leica Aperio Versa 200. Immunohistochemical staining was blindly scored by two pathologists, and the average score was taken as the final result. The percentage of positive tumor cells was determined in at least five areas and scored as the following: 0, ≤5%; 1, 5–25%; 2, 25–50%; 3, 50–75%; 4, ≥75%. The intensity of staining in positive cells was scored as 1 (weak), 2 (moderate), or 3 (intense). The total score was calculated by multiplying the scores for percentage and the intensity of staining.

### 2.15. Statistical Analysis

All statistical analyses and graphical presentations were performed using GraphPad Prism 8.0. Data were shown as mean ± standard error of the mean (SEM). Two sample t-tests were used to compare two independent groups, and ANOVA was used to compare multiple comparisons. The difference is considered significant at *p* < 0.05. Each experiment was repeated at least 3 times, and representative experiments are shown.

## 3. Results

### 3.1. Exo70 Expression Was Clinically Associated with Innate Platinum Resistance in EOC

**Figure 1:**
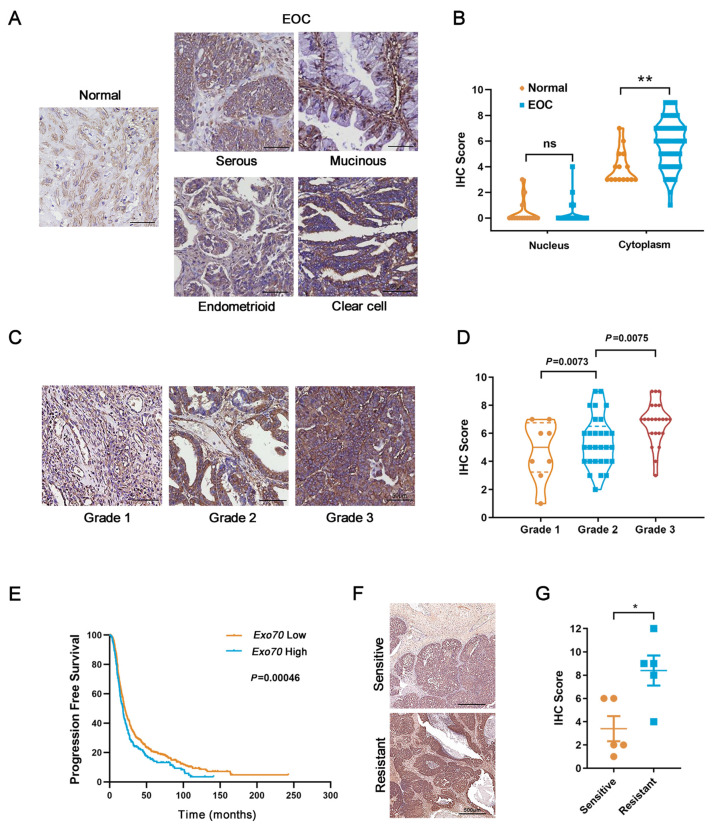
Exo70 is overexpressed in epithelial ovarian cancer (EOC) patients’ tissues and associated with shortened progression-free survival in platinum-treated EOC patients. (**A**) Representative photographs of immunohistochemistry (IHC) for Exo70 in normal ovarian and EOC tissues. Scale bar, 50 μm. (**B**) Statistics of Exo70 expression level in normal ovarian and EOC tissues. ** *p* < 0.01. (**C**) Representative photographs of immunohistochemistry for Exo70 in different pathological grades of EOC tissues. Scale bar, 50 μm. (**D**) Statistics of Exo70 expression level in different pathological grades of EOC tissues. Bonferroni correction was used to adjust *p* values. (**E**) Progression-free survival curve in platinum treated EOC patients of high versus low Exo70 expression. Data were generated online by using the Kaplan-Meier Plotter database based on microarray gene expression data from patients for whom PFS data are available (www.kmplot.com/ovarian (accessed on 3 February 2019)). (**F**) IHC staining of Exo70 in samples from platinum-sensitive (upper panel) and platinum-resistant (lower panel) EOC patients. Scale bar, 500 μm. (**G**) Statistics of Exo70 expression level in different pathological grades of EOC tissues. * *p* < 0.05.

To evaluate the potential role of Exo70 in EOC progression, we first conducted bioinformatics analyses using Oncomine and UALCAN databases. The results showed that Exo70 was highly expressed in EOC tissues of different pathological types compared to normal ovarian tissues, and the mRNA expression level of Exo70 was positively correlated with the pathological grade of EOC (Figure S1A–C). We then carried out immunohistochemistry (IHC) with anti-Exo70 antibody in clinical samples. Expression of Exo70 was mainly in the cytoplasm. The cytoplasmic expression level of Exo70 was generally higher in EOC tissue samples than in normal tissues (Figure 1A,B), and the expression level of Exo70 gradually increased in the EOC tissue samples with the increase in pathological grade (Figure 1C,D).

Since platinum resistance is a major hindrance in EOC treatment, we further investigated the relationship between Exo70 expression and innate platinum resistance of EOC. Kaplan-Meier survival analysis (Kaplan-Meier plotter database) indicated that the progression-free survival of EOC patients treated with platinum drugs was significantly shortened in the highly-expressing Exo70 mRNA group (*p* = 0.00046, Figure 1E). Furthermore, we collected tumor tissue samples from 10 EOC patients who had received surgical resection combined with platinum-based chemotherapy as initial treatment and divided them into innate platinum-sensitive and innate platinum-resistant groups according to the platinum-free interval (PFI). Immunohistochemistry results showed that the protein levels of Exo70 in the platinum-sensitive group were generally lower than that of the platinum-resistant group (Figure 1F,G). The described results thus suggest the clinical correlation of Exo70 expression with innate platinum resistance and the potential use of Exo70 expression as an indicator of cisplatin sensitivity in EOC patients.

### 3.2. Exo70 Suppressed Cisplatin Sensitivity of EOC Cells In Vitro and In Vivo

**Figure 2:**
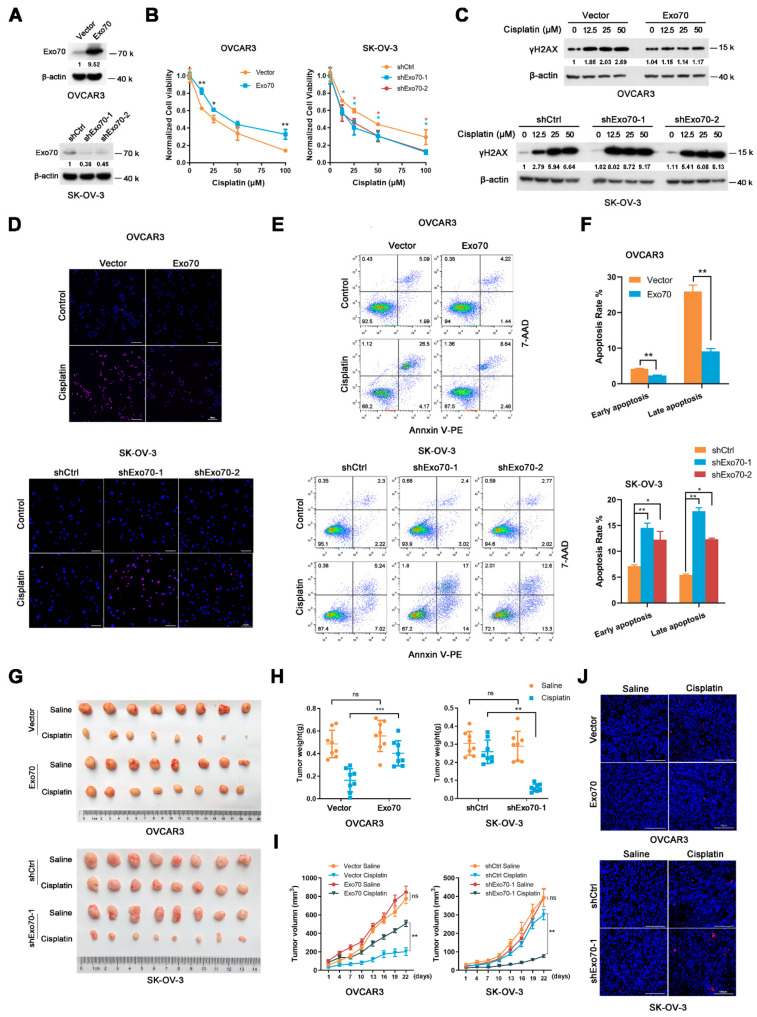
Exo70 affects cisplatin sensitivity of EOC in vitro and in vivo. (**A**) Western blotting showed the overexpression of Exo70 in OVCAR3 cells and the knockdown of Exo70 by shRNA in SK-OV-3 cells. (**B**) CCK8 assay examined the cytotoxicity of cisplatin in OVCAR3 cells (left panel) and SK-OV-3 cells (right panel). * *p* < 0.05, ** *p* < 0.01. (**C**) Western blotting showed γH2AX expression level in OVCAR3 cells or SK-OV-3 cells after 24 h treatment with cisplatin. (**D**) Representative images of TUNEL staining for OVCAR3 cells or SK-OV-3 cells. Cells were treated with 50 μM cisplatin for 3 h. Scale bar, 100 μm. (**E**) Representative images of FACS analysis by Annexin V and 7-AAD double staining. (**F**) Percentage of apoptotic cells as detected by FACS analysis. Data were expressed as mean ± SEM, *n* = 3. * *p* < 0.05, ** *p* < 0.01 (**G**) Pictures of OVCAR3 or SK-OV-3 cells’ xenograft tumors in nude mice treated with or without cisplatin. (**H**) Tumor weights of mice with or without cisplatin treatment. Data were expressed as mean ± SEM, *n* = 8. ns: not significant, ** *p* < 0.01, *** *p* < 0.001 (**I**) Growth curve of tumor volume in mice with or without cisplatin treatment. Data were expressed as mean ± SEM. ns: not significant, ** *p* < 0.01 (**J**) Representative images of TUNEL staining of tumors with or without cisplatin treatment. Scale bar, 100 μm.

To further investigate the possible role of Exo70 in promoting cisplatin resistance in EOC, two EOC cell lines with different basal expression levels of Exo70, OVCAR3 (low expression) and SK-OV-3 (high expression) were selected (Figure S2A,B). Neither overexpression of Exo70 in OVCAR3 cells nor Exo70 knockdown in SK-OV-3 cells affected their growth or survival (Figure 2A and Figure S2C,D); however, overexpression of Exo70 in OVCAR3 cells reduced while knockdown of Exo70 in SK-OV-3 cells enhanced their sensitivity to cisplatin treatment (Figure 2B). Moreover, DNA damage induced by cisplatin, as indicated by increased accumulation of γH2AX, was decreased in Exo70-overexpressed OVCAR3 cells and increased in Exo70-knockdown SK-OV-3 cells (Figure 2C). TUNEL and Annexin V/7-AAD double-staining assays also indicated the inhibitory effect of Exo70 on cisplatin-induced apoptosis of EOC cells (Figure 2D,F). These in vitro results were further verified in vivo with a subcutaneous xenograft tumor model in nude mice, in which Exo70 greatly suppressed the chemotherapeutic effect of cisplatin (Figure 2G–J). Therefore, our data indicated that Exo70 promoted cisplatin resistance of EOC cells.

### 3.3. Exo70 Accelerated the Efflux of Cisplatin in EOC Cells through Exocytosis

**Figure 3:**
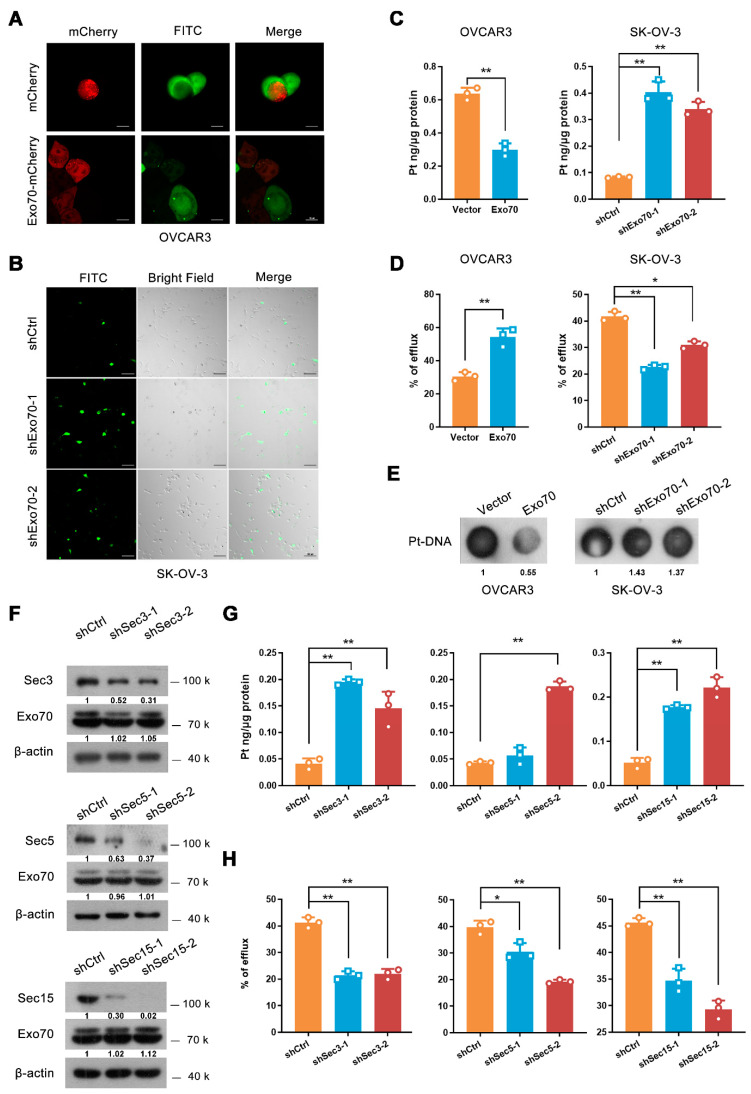
Exo70 reduces the intracellular accumulation of cisplatin through exocytosis. (**A**) Representative images of OVCAR3 cells treated with FITC-cisplatin. OVCAR3 cells expressing mCherry or mCherry-Exo70 were mixed with unlabeled OVCAR3 cells, incubated with FITC-cisplatin for 3 h, and then visualized by confocal microscopy. Scale bar, 10 μm. (**B**) Representative images of SK-OV-3 cells treated with FITC-cisplatin. SK-OV-3 cells were cultured with FITC-Cisplatin for 3 h and then visualized by confocal microscopy. Scale bar, 100 μm. (**C**) ICP-MS showed the intracellular cisplatin accumulation in OVCAR3 and SK-OV-3 cells. Data were expressed as mean ± SEM, *n* = 3. ** *p* < 0.01. (**D**) ICP-MS evaluated the percentage of extracellular cisplatin in OVCAR3 and SK-OV-3 cells. Data were expressed as mean ± SEM, *n* = 3. * *p* < 0.05, ** *p* < 0.01. (**E**) Dot-blot detected cisplatin-DNA adduct in genomic DNA in OVCAR3 and SK-OV-3 cells. (**F**) Western blotting showed shRNA-mediated knockdown of Sec3, Sec5, and Sec15 in SK-OV-3 cells. (**G**,**H**) ICP-MS was used to detect the intracellular cisplatin accumulation (**G**) and percentage of extracellular cisplatin (**H**) in SK-OV-3 cells. Data were expressed as mean ± SEM, *n* = 3. * *p* < 0.05, ** *p* < 0.01.

We next explored how Exo70 regulated the tolerance of EOC cells to cisplatin-induced damage. By fluorescence titration assay, we first excluded the possibility that Exo70 directly bound cisplatin to reduce its activity (Figure S3A). Since Exo70 is a key subunit of the exocyst complex essential for exocytosis, we assumed that Exo70 might increase the cisplatin resistance of EOC cells by promoting cisplatin efflux. Fluorescent cisplatin (FITC-cisplatin, F-DDP) was synthesized and applied to EOC cells. Overexpression of Exo70 significantly reduced the intracellular fluorescent intensity of cisplatin in OVCAR3 cells (Figure 3A), while knockdown of Exo70 strongly enhanced that in SK-OV-3 cells (Figure 3B). This observation was then verified through ICP-MS. Overexpression of Exo70 in OVCAR3 cells decreased the intracellular accumulation of cisplatin while increasing the extracellular cisplatin concentration. On the contrary, Exo70 knockdown in SK-OV-3 cells exhibited promoting and suppressing effects on intracellular and extracellular cisplatin concentrations, respectively (Figure 3C,D). Consistently, the dot-blot experiment further showed that altering the expression level of Exo70 could negatively influence the formation of Pt (platinum)-DNA cross-links in EOC cells upon cisplatin treatment (Figure 3E). These results indicated that Exo70 inhibited the response of EOC cells to cisplatin via promoting cisplatin efflux.

As Exo70 is a critical subunit of the exocyst, we investigated whether the effect of Exo70 on platinum efflux depended on exocytosis. Results from ICP-MS showed that similar to the knockdown of Exo70, silencing of other components (Sec3, Sec5, or Sec15) also led to the increased intracellular cisplatin accumulation and the reduced extracellular cisplatin concentration (Figure 3F–H). We then fractionated the components (large vesicles, small vesicles, and supernatant) of the extracellular fluid of EOC cells [29], and found that cisplatin was predominantly present in the supernatant (Figure S3B), suggesting that secretory exocytosis, instead of exosome or other vesicle transports, is the main avenue for EOC cells to discharge cisplatin. ATP7A and ATP7B are well-known copper transporters associated with platinum efflux and platinum resistance [16,17,18,30]. We next investigated if ATP7A/B involved in Exo70 regulated cisplatin exclusion. Overexpression Exo70 did not alter the mRNA expression of ATP7A/7B (Figure S3C). Additionally, knockdown of ATP7A and/or ATP7B, could not impair the effect of Exo70 on cisplatin effluence (Figure S3D,E), suggesting that Exo70 reduced intracellular accumulation of cisplatin independent of classic copper transporters.

### 3.4. Cisplatin-Induced Exo70 Degradation Was Hampered in Acquired Cisplatin-Resistant Cells

**Figure 4:**
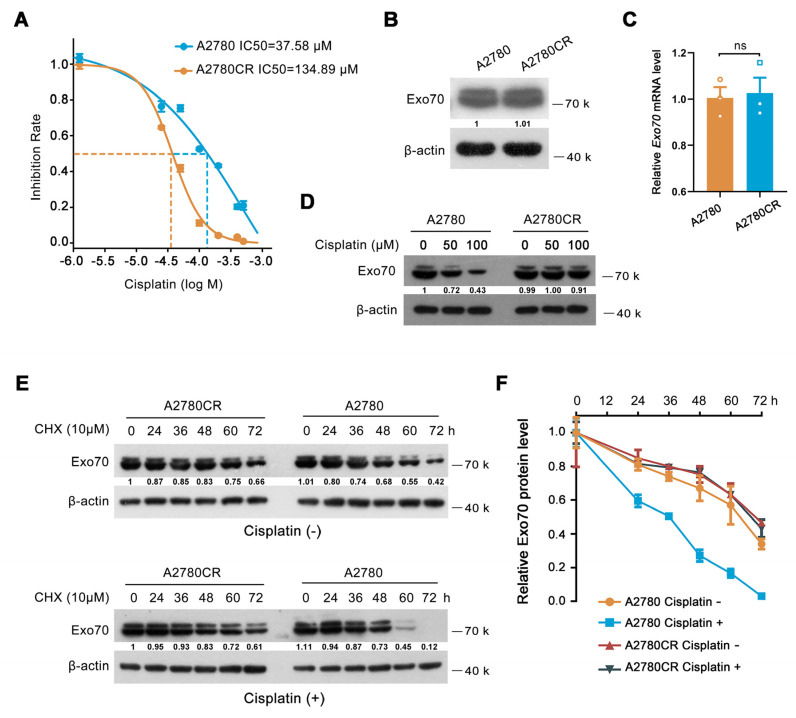
Exo70 is stabilized in cisplatin−resistant EOC cells. (**A**) IC50s of cisplatin in A2780 and A2780CR cells were measured by CCK8 assay. (**B**) Western blotting showed Exo70 expressions in A2780 and A2780CR cells. (**C**) RT−PCR results showed the Exo70 mRNA expression levels in A2780 and A2780CR cells. ns: not significant. (**D**) Western blotting detected Exo70 expression in A2780 and A2780CR cells with the treatment of different doses of cisplatin for 3 h. (**E**) Western blotting detected the half−lives of Exo70 in A2780 and A2780CR cells. Cells were treated with 10 μM CHX with or without 12.5 μM cisplatin for indicated times. (**F**) Density analysis of Exo70 expressing level with CHX treatment together with or without cisplatin in A2780 or A2780CR cells. Data were presented as mean ± SEM, *n* = 3.

Previously described results have demonstrated that increased Exo70 expression level was correlated with innate cisplatin resistance of EOC, and Exo70 suppressed cisplatin sensitivity via accelerating exocytosis-mediated efflux of cisplatin in EOC cells. We then studied whether Exo70 also participated in the acquired cisplatin resistance of EOC cells. A cisplatin-resistant cell line A2780CR was established by exposing A2780 cells (cisplatin-sensitive) to high-dose cisplatin shock following low-dose cisplatin maintenance (Figure 4A). Unexpectedly, both the mRNA and protein levels of Exo70 in A2780CR cells were comparable to that in A2780 cells (Figure 4B,C). These results were further confirmed by the data from the GEO database, in which no difference of Exo70 mRNA levels was observed between cisplatin-sensitive and cisplatin-resistant A2780 cells (Figure S4A). However, a fascinating phenomenon was observed that cisplatin dose-dependently induced degradation of Exo70 protein, without reducing its mRNA expression, in A2780 cells but not A2780CR cells (Figure 4D–F and Figure S4B). These results suggested that in sensitive cells cisplatin-induced Exo70 degradation in order to reduce its counteractive effect, which was impaired and probably led to acquired resistance of EOC cells to cisplatin.

### 3.5. Autophagy-Lysosome Pathway Mediated Cisplatin-Induced Exo70 Degradation

**Figure 5:**
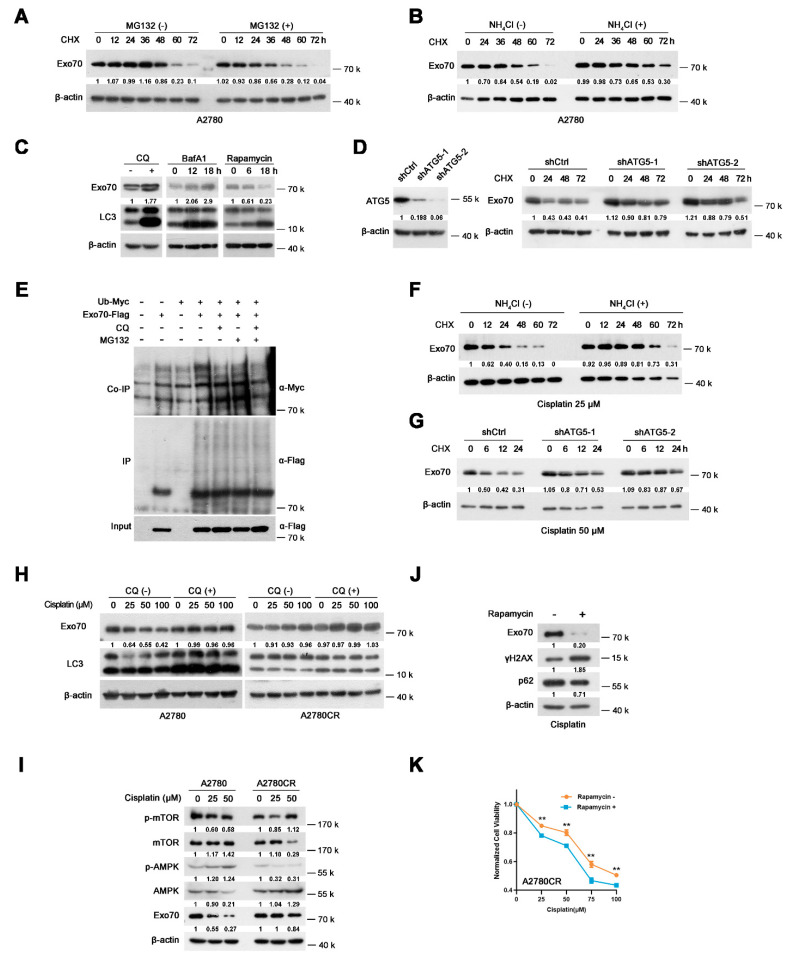
Cisplatin induces autophagy−lysosomal degradation of Exo70 in cisplatin-sensitive EOC cells, but not in cisplatin−resistant cells. (**A**) Western blotting showed the half−lives of Exo70 in A2780 cells with or without 20 μM MG132 treatments. (**B**) Western blotting showed the half−lives of Exo70 in A2780 cells with or without 10 mM NH4Cl treatments. (**C**) Western blotting showed the expression of Exo70 and LC3 in A2780 cells treated with 20 μM CQ, 200 nM BafA1, or 20 μM rapamycin for the indicated time. (**D**) Western blotting showed the effect of shRNA targeting ATG5 on the half−lives of Exo70. (**E**) The Co−IP assay showed the ubiquitin levels of Exo70 inA2780 cells treated with different reagents. (**F**) Western blotting showed the half−lives of Exo70 in A2780 cells with 25 μM cisplatin treatment with or without 10 mM NH4Cl. (**G**) Western blotting showed the half−lives of Exo70 in ATG5 control and knockdown A2780 cells, which were treated with 50 μM cisplatin. (**H**) Western blotting showed the expression of Exo70 and LC3 in A2780 cells or A2780CR cells treated with indicated doses of cisplatin. (**I**) Western blotting showed the expression of p−mTOR, mTOR, p−AMPK, and AMPK in A2780 and A2780CR cells under the treatment of the indicated dose of cisplatin for 24 h. (**J**) Western blotting showed the expression of Exo70, γH2AX, and p62 proteins in A2780CR cells upon treatment with 100 μM cisplatin in the presence or absence of 20 mM rapamycin. (**K**) CCK8 assay was carried out to examine the cytotoxicity of cisplatin in A2780CR cells with or without rapamycin (20 mM) treatment. Data were presented as mean ± SEM, *n* = 4. ** *p* < 0.01.

To study how EOC cells gain resistance to cisplatin-induced Exo70 degradation during repetitive cisplatin treatments, we first explored the mechanisms involved in Exo70 degradation in cisplatin-sensitive EOC cells. Lysosomal inhibitor NH_4_Cl, but not proteasome inhibitor MG132, extended the half-life of Exo70 (Figure 5A,B and Figure S5A,B), suggesting that the degradation of Exo70 is mediated by lysosomes rather than proteasomes. We further found that autolysosome inhibitors chloroquine (CQ) and bafilomycin A1 (BafA1) increased while the autophagy inducer rapamycin decreased the protein level of Exo70 in A2780 cells (Figure 5C). Moreover, the knockdown of autophagy-related protein ATG5 lengthened the half-time of Exo70 protein (Figure 5D). These results indicated that the autophagy-lysosome pathway was involved in Exo70 degradation. However, CQ treatment did not elevate the ubiquitination of Exo70 protein (Figure 5E), suggesting that Exo70 might be degraded via the non-selective autophagy-lysosome pathway in cisplatin-sensitive EOC cells.

We then investigated how cisplatin accelerated Exo70 degradation in sensitive but not resistant cells. Under cisplatin treatment, NH_4_Cl or knockdown of ATG5, instead of MG132, extended the half-life of Exo70 (Figure 5F,G and Figure S5C), suggesting that cisplatin-induced Exo70 degradation was also through the autophagy-lysosomal pathway. We further found that CQ blocked cisplatin-induced Exo70 degradation, but showed no significant influence on A2780CR cells (Figure 5H). In addition, cisplatin-induced AMPK phosphorylation and mTOR dephosphorylation in A2780 cells but not A2780CR cells, which were conducive to active autophagy (Figure 5I). These results thus indicated that cisplatin-induced autophagy-lysosome-mediated degradation of Exo70 protein through regulating phosphorylation of AMPK and mTOR. This degradation was impaired and lead to cisplatin resistance in the acquired resistant cells. Moreover, rapamycin, a well-known autophagy activator by inhibiting mTOR phosphorylation, could accelerate Exo70 degradation and effectively reverse the acquired resistance of A2780CR cells (Figure 5J,K).

### 3.6. Targeting Exo70 with shRNA or Small Molecule Reversed Acquired Cisplatin Resistance of EOC Cells

**Figure 6:**
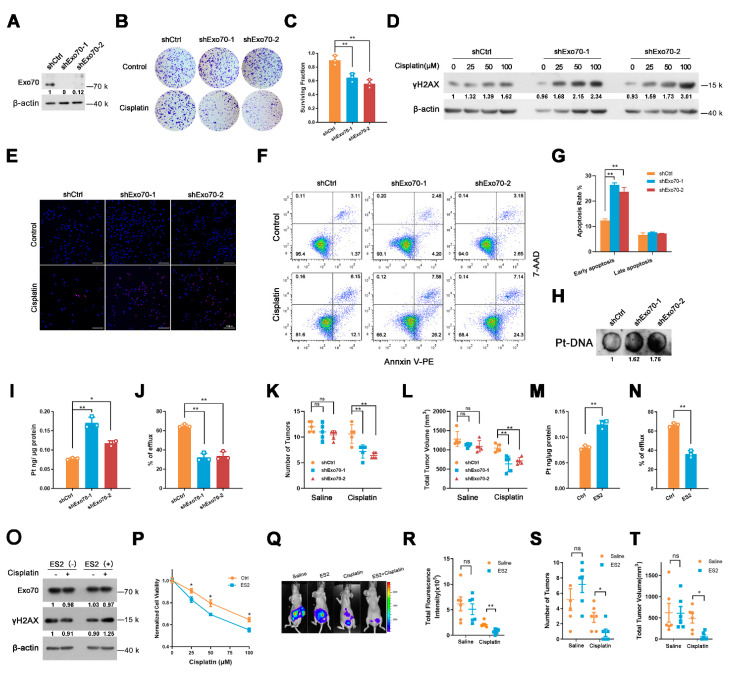
Exo70 silencing or inhibition of its activity reverses cisplatin resistance on A2780CR cells. (**A**) Western blotting showed the effects of Exo70 knockdown by shRNA in A2780CR cells. (**B**) Representative photos of the colony−formation assay showed the cytotoxicity of cisplatin on A2780CR cells. (**C**) Percentage of cells survived in colony formation assay. Data were expressed as mean ± SEM, *n* = 3. ** *p* < 0.01 (**D**) Western blotting showed γH2AX expression in A2780CR cells with 100 μM cisplatin treatment for 3 h. (**E**) Representative images of TUNEL assay showed the apoptotic cells after cisplatin treatment. Scale bar, 100 μm. (**F**) Representative images of FACS analysis by Annexin V and 7−AAD double staining. (**G**) Percentage of apoptotic cells as detected by FACS analysis. Data were expressed as mean ± SEM, *n* = 3. ** *p* < 0.01 (**H**) Dot−blot assay showed the cisplatin−DNA adduct in genomic DNA in A2780CR cells. (**I**,**J**) ICP-MS results showed the intracellular cisplatin accumulation (**I**) and extracellular percentage of cisplatin (**J**) in A2780CR cells. Data were expressed as mean ± SEM, *n* = 3. * *p* < 0.05, ** *p* < 0.01 (**K**) The number of tumors in mice with peritoneal tumor xenograft with or without cisplatin treatment. Data were expressed as mean ± SEM, *n* = 5. ** *p* < 0.01. (**L**) Total tumor volume in mice with peritoneal tumor xenograft with or without cisplatin treatment. Data were expressed as mean ± SEM, *n* = 5. ** *p* < 0.01 (**M**,**N**) ICP-MS was carried out to detect the intracellular cisplatin accumulation (**M**) and extracellular percentage of cisplatin (**N**) in A2780CR cells treated with or without ES2. Data were expressed as mean ± SEM, *n* = 3. ** *p* < 0.01. (**O**) Western blotting showed γH2AX expression in A2780CR cells with 100 μM cisplatin together with ES2. (**P**) CCK8 assay accessed the cytotoxicity of cisplatin with or without ES2 treatment. Data were expressed as mean ± SEM, *n* = 4. * *p* < 0.05. (**Q**) Bioluminescent imaging of the nude mice bearing A2780CR tumors. (**R**) Quantitative analysis of tumors by measurement of luminescence in nude mice bearing A2780CR tumors. Data were expressed as mean ± SEM, *n* = 6. ns: not significant, ** *p* < 0.01. (**S**) The number of tumors in mice bearing A2780CR tumors. Data were expressed as mean ± SEM, *n* = 5. * *p* < 0.05. (**T**) Total tumor volume in mice bearing A2780CR tumors. Data were expressed as mean ± SEM, *n* = 5. * *p* < 0.05.

We next investigated whether targeting Exo70 with shRNA or small molecule could be an effective way to reverse the acquired cisplatin resistance of EOC cells. As shown in Figure 6A–D, knockdown of Exo70 by shRNA greatly enhanced the capacity of cisplatin to reduce the colony formation ability and to increase γH2AX accumulation of A2780CR cells. TUNEL, Annexin V/7-AAD double staining, and Pt-DNA cross-linking assays also showed that knockdown of Exo70 increased the cytotoxic effects of cisplatin (Figure 6E–H). ICP-MS results further demonstrated that Exo70 knockdown in A2780CR cells increased intracellular cisplatin accumulation and reduced extracellular cisplatin concentration (Figure 6I,J). The improving effect of Exo70 knockdown on the cisplatin sensitivity of A2780CR cells was further verified in vivo by a nude mouse xenograft tumor model (Figure 6K,L). We then studied if inhibition of Exo70 function could also reverse the drug resistance in A2780CR cells. ES2 is a functional inhibitor of exocytosis by directly binding Exo70 [31]. Similar to knockdown of Exo70, ES2 treatment could increase the intracellular cisplatin accumulation, promote cisplatin-induced γH2AX accumulation, and enhance the cytotoxicity of cisplatin in A2780CR cells both in vitro and in vivo (Figure 6M–T). These results indicated that targeting Exo70 effectively improved the cisplatin sensitivity of acquired cisplatin-resistant EOC cells, thus provided a new target and potential drugs for the treatment of cisplatin-resistant EOC.

## 4. Discussion

Platinum drugs are currently first-line chemotherapy drugs for ovarian cancer. However, 80% of patients with advanced EOC relapse and become platinum-resistant, thereafter with limited effective treatment options and a low 5-year survival rate [3]. There is an urgent unmet need to understand molecules driving platinum resistance in EOC so that better therapeutic strategies and predictive biomarkers may be developed to improve outcomes of this fatal disease. Among the reported mechanisms involved in platinum resistance, the decreased intracellular accumulation of platinum drugs is a major mechanism leading to chemotherapy resistance. In this study, we reported that Exo70, a member of the exocyst, promoted cisplatin resistance through facilitating exocytosis-mediated cisplatin efflux in EOC cells (Figure 2 and Figure 3). The expression level of Exo70 is clinically associated with innate cisplatin resistance of EOC (Figure 1), suggesting that Exo70 may help to guide treatment decisions, i.e., whether an individual would respond to platinum-based chemotherapy as initial treatment. We further found that cisplatin-induced autophagy-lysosome degradation of the Exo70 protein was hampered in acquired cisplatin-resistant EOC cells (Figure 4 and Figure 5), suggesting that testing the half-life of the Exo70 protein under cisplatin treatment in patient-derived tumor cells could be a promising approach allowing us to predict whether an EOC patient could still respond to platinum-based chemotherapy after relapse. We also discovered that Exo70 inhibitor ES2 could enhance the cytotoxicity of cisplatin in SK-OV-3 cells with innate cisplatin resistance (Figure S4C,D). Exo70 knockdown and ES2 effectively reversed the acquired cisplatin resistance of EOC cells in vitro and in vivo (Figure 6). Although future works might be required to validate potential side effects, targeting Exo70 would be a novel therapeutic strategy to overcome cisplatin resistance in EOC treatment. Therefore, our study provided a predictive factor, therapeutic target, and lead compound for innate and acquired cisplatin resistance in EOC treatment, which may benefit the outcomes of this refractory disease.

As a critical member of the exocyst, Exo70 was reported to promote invasion via enhancing MMPs secretion in tumor cells [25,26,32], and increased glucose uptake by accelerating inter-endosomal GLUT4 traffic to the plasma membrane in adipocytes [33,34]. In the present study, we reported for the first time that Exo70 promotes cisplatin efflux in EOC cells. To understand how Exo70 facilitates cisplatin efflux, we knocked down several other components of the exocyst, including Sec3, Sec5, and Sec15, and found that they were also involved in the efflux of cisplatin (Figure 3F–H), which meant the whole exocyst complex mediated cisplatin efflux. Future studies might be required to validate the role of other exocyst members in cisplatin resistance. Moreover, most of the discharged cisplatin was in the supernatant rather than extracellular vesicles (Figure S3B), suggesting that extracellular vesicle is not the major form for cisplatin efflux. Although existing literature reports indicate that ATP7A and ATP7B, are critical for the cisplatin resistance of cancers [16,17]. However, this ATP7A/ATP7B is not utilized by Exo70 to promote cisplatin efflux (Figure S3C–E). Lysosomal exocytosis is a reported mechanism to accelerate the clearance of cisplatin accumulated in lysosomes [19]; interestingly, Exo70 is also reported to enhance polarized lysosome secretion at the immune synapse [23]. We, therefore, speculated that Exo70 may facilitate the exocytosis of cisplatin-loaded lysosomes to induce cisplatin resistance.

Different from the innate cisplatin resistance which was correlated with the expression level of Exo70 (Figure 1), the acquired cisplatin resistance was at least partially attributed to the blockade of cisplatin-induced degradation of Exo70 protein (Figure 4). Therefore, correcting the over-stability of Exo70 protein in response to cisplatin might overcome the acquired cisplatin resistance. We further found that cisplatin-induced AMPK phosphorylation and mTOR dephosphorylation activate autophagy-lysosome degradation of Exo70 in A2780 cells but not A2780CR cells (Figure 5F–I). Rapamycin, a well-known autophagy activator by inhibiting mTOR phosphorylation, could accelerate Exo70 degradation (Figure 5J), and effectively reversed the acquired resistance of A2780CR to cisplatin (Figure 5K). Consistent with our study, a previous report has indicated that rapamycin enhanced the antitumor efficacy of oxaliplatin in cisplatin-resistant ovarian cancer cells [35].

Although autophagy in drug resistance has been extensively studied in different cancers, its roles vary. Consistent with our results, a previous report showed that long-term cisplatin exposure impaired autophagy and thus caused cisplatin resistance in lung cancer cells [36]. Low level of autophagy is also reported to facilitate ovarian cancer progression and upregulated autophagy is more sensitive to chemotherapy [37]. However, other reports also indicated that autophagy plays a cytoprotective role against cisplatin in EOC cells [38,39]. As a lysosomal degradation process to engulf unwanted proteins and organelles, the roles of autophagy in cancer developments are complicated and context-dependent. Our study demonstrated that in cisplatin-sensitive cells, the activation of autophagy further degraded Exo70 to increase cisplatin intracellular storage, thus forming a positive feedback loop to maximize the cytotoxic effect of cisplatin. However, during prolonged cisplatin treatment, the autophagy lysosomal degradation process of Exo70 was hampered and Exo70 was stabilized, which in turn reduced intracellular cisplatin storage, resulting in a negative feedback loop for cells to gain acquired cisplatin resistance. The mechanisms involved in the blockade of autophagy in cisplatin-resistant EOC cells need to be further studied. It was reported that prolonged treatment of cisplatin could upregulate some autophagy suppressor genes, such as BCl-2 to suppress autophagy [40]. Similarly, our result also showed that cisplatin significantly upregulated Bcl-2 in A2780CR cells, which might be a mechanism involved in the insensitivity of A2780CR to cisplatin-induced autophagy activation (Figure S5D).

## 5. Conclusions

In summary, our results suggest that Exo70 accelerates exocyst-mediated cisplatin efflux to induce cisplatin resistance in EOC cells, the blockade of the autophagy-lysosome degradation process of Exo70 leads to acquired cisplatin resistance, inhibiting Exo70 could reverse innate and acquired cisplatin resistance in EOC. This study provides new insight into the mechanism of platinum drug resistance in EOC and identifies Exo70 as a potential prognostic factor for platinum-based chemotherapy and a new target to reverse innate and acquired cisplatin resistance in EOC treatment.

## Data Availability

Kaplan-Meier Plotter database, Oncomine database and UALCAN database were respectively available on platforms (www.kmplot.com/ovarian (accessed on 3 February 2019), https://www.oncomine.org/ (accessed on: 21 January 2019) and http://ualcan.path.uab.edu/ (accessed on: 24 January 2019)).

## Supplementary Materials

The following are available online at https://www.mdpi.com/article/10.3390/cancers13143467/s1, Figure S1: EXOC7 was highly expressed in EOC samples; Figure S2: Exo70 expression did not affect the growth ratio of EOC cells; Figure S3: Exo70 efflux cisplatin to supernatant independent of ATP7A and ATP7B; Figure S4: Exo70 affected the sensitivity of cells to cisplatin; Figure S5: Autophagy affected the protein stability of Exo70; Table S1: Information of antibodies; Table S2: Sequences of oligonucleotide primers for shRNA construction; Table S3: Sequences of primers for real-time PCR; Table S4: Characteristics of the clinical ovarian cancer patients; Figure S6: Quantification of Western Blot; Figure S7:Original images for blots/gels, Figure S8: STR profiling reports of cell lines.
