# Supplementary material for: Deregulation of Exo70 Facilitates Innate and Acquired Cisplatin Resistance in Epithelial Ovarian Cancer by Promoting Cisplatin Efflux
Source: Cancers (Basel). 2021 Jul 11;13(14):3467. doi: 10.3390/cancers13143467 (PMC8304026; doi:10.3390/cancers13143467)

# Figure 2.

①

OVCAR3

vector Exo70

上 Exo70

下 Actin

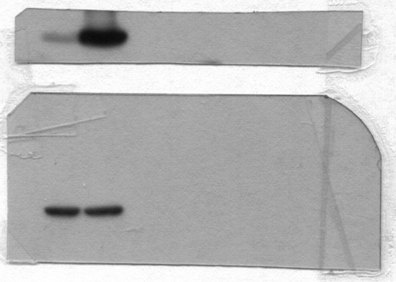

②

SK-OV-3

shCtrl shExo70-1 shExo70-2

上 Exo70

下 Actin

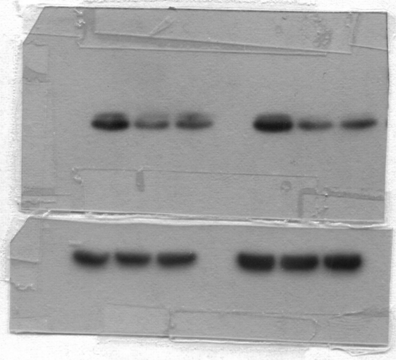

③

OVCAR3

Vector Exo70

Cisplatin(uM) 0 12.5 25 50 0 12.5 25 50

上 rH2AX

中 Actin

下 Exo70

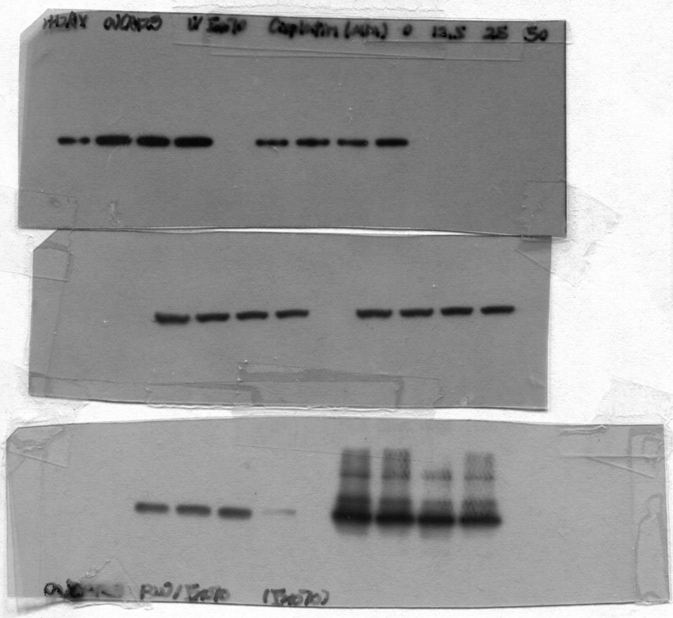

④

SK-OV-3

shCtrl shExo70-1 shExo70-2

Cisplatin(uM) 0 12.5 25 50 0 12.5 25 50 0 12.5 25 50

上 Exo70

中 rH2AX

下 Actin

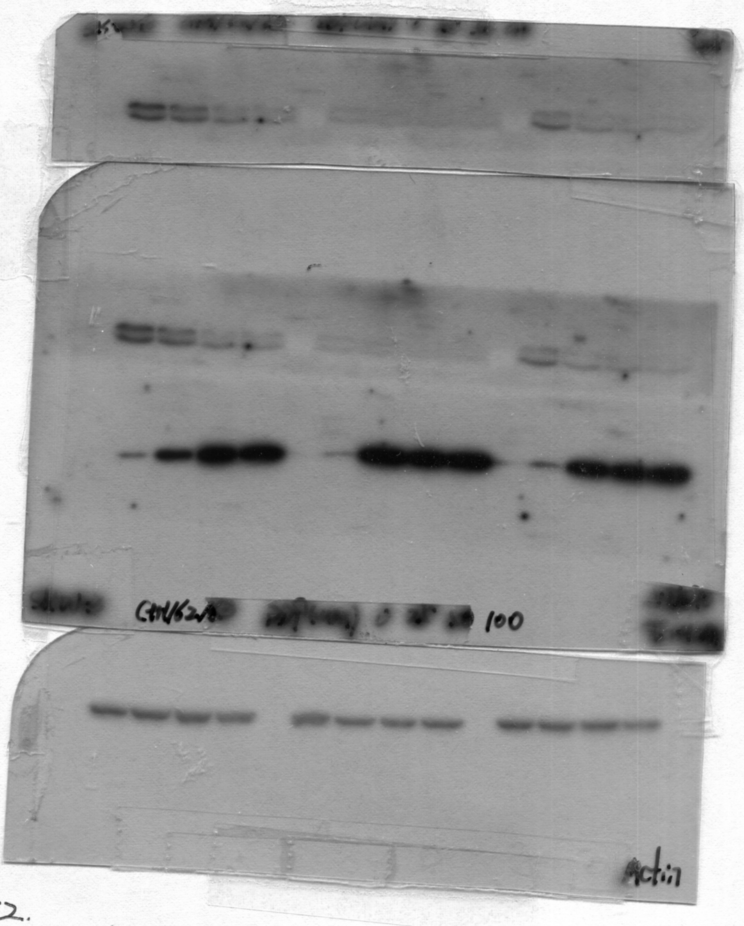


# Figure S2.

OVCAR3 SKOV-3 左 Exo70 右 Actin

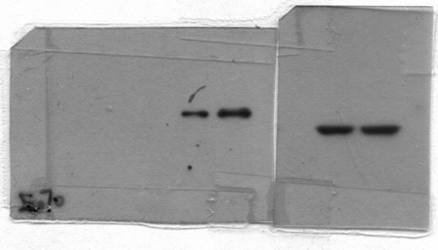

Figure3

① Dot Blot

左 OVCAR3 ; 右 SK-OV-3

Vetor Exo70 shCtrl shExo70-1 shExo70-2

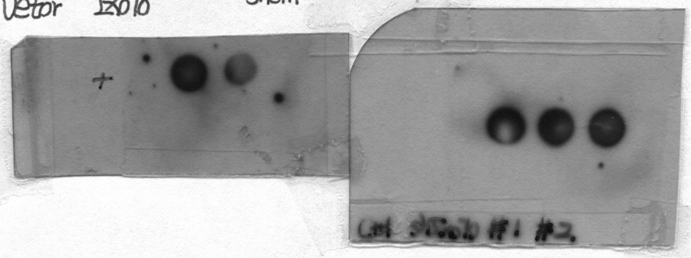


② 左→右 上↓下

Sec15, Sec3, sec5 目标蛋白, Exo70, Actin

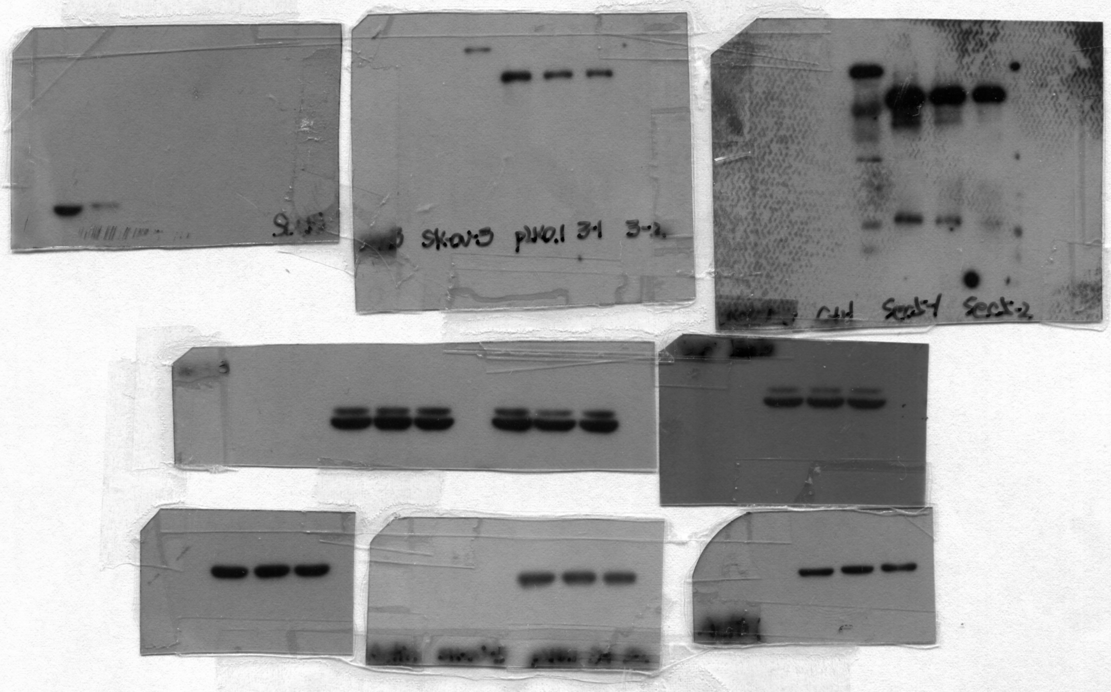


Figure S3

③ 左ATP7A | 右ATP7B

Actin

左7A 右7B

ATP7A →

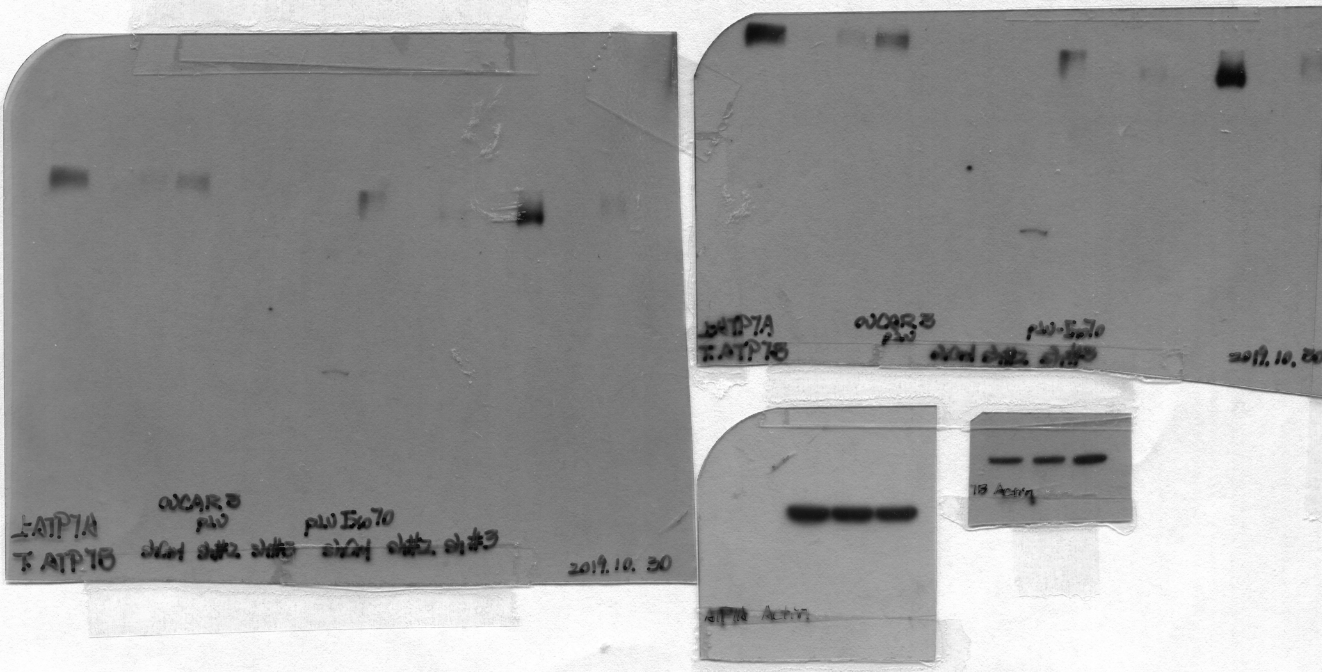

Figure 4

① A2780 A2780Cis 左Exo70 右Actin

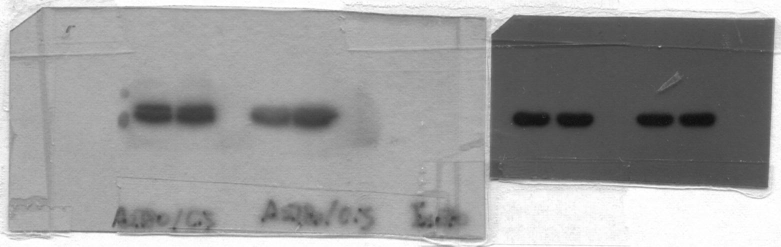

② A2780 A2780Cis

Cisplatin(uM) 0 50 100 ; 0 50 100

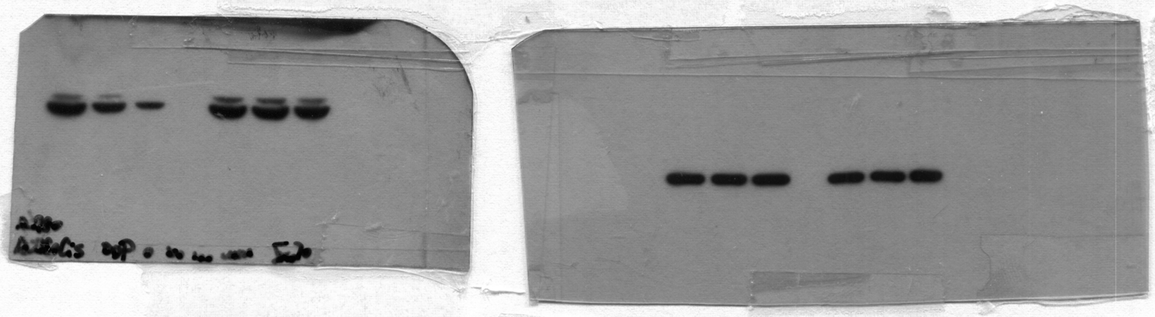

③ A2780Cis / A2780

CHX(uM) 0 24 36 48 60 72 h.

上Exo70

下Actin

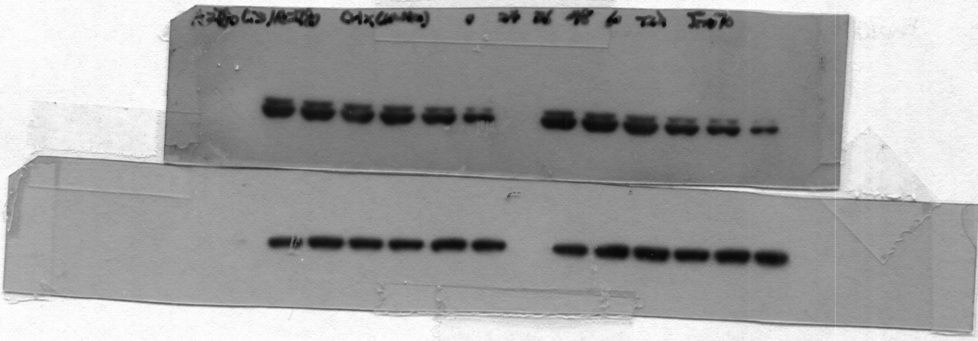

④ A2780Cis / A2780

CHX(uM) 0 24 36 48 60 72h

Cisplatin 12.5 uM

上 Exo70

下 Actin

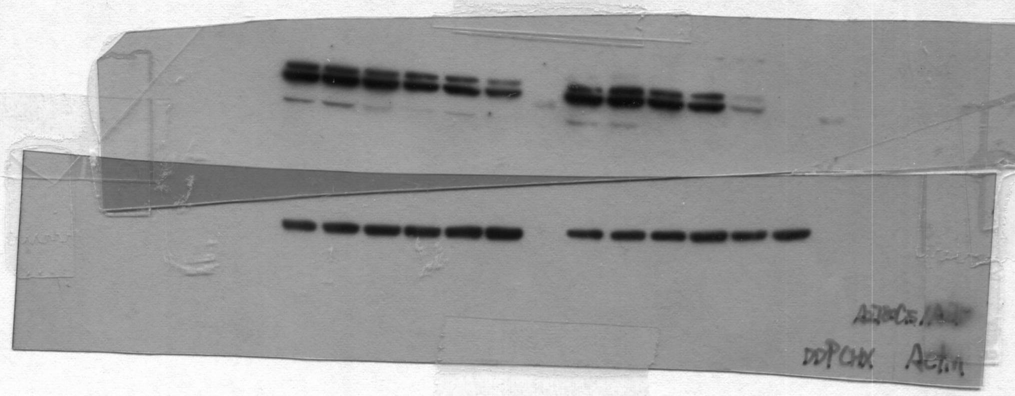

Fig S5

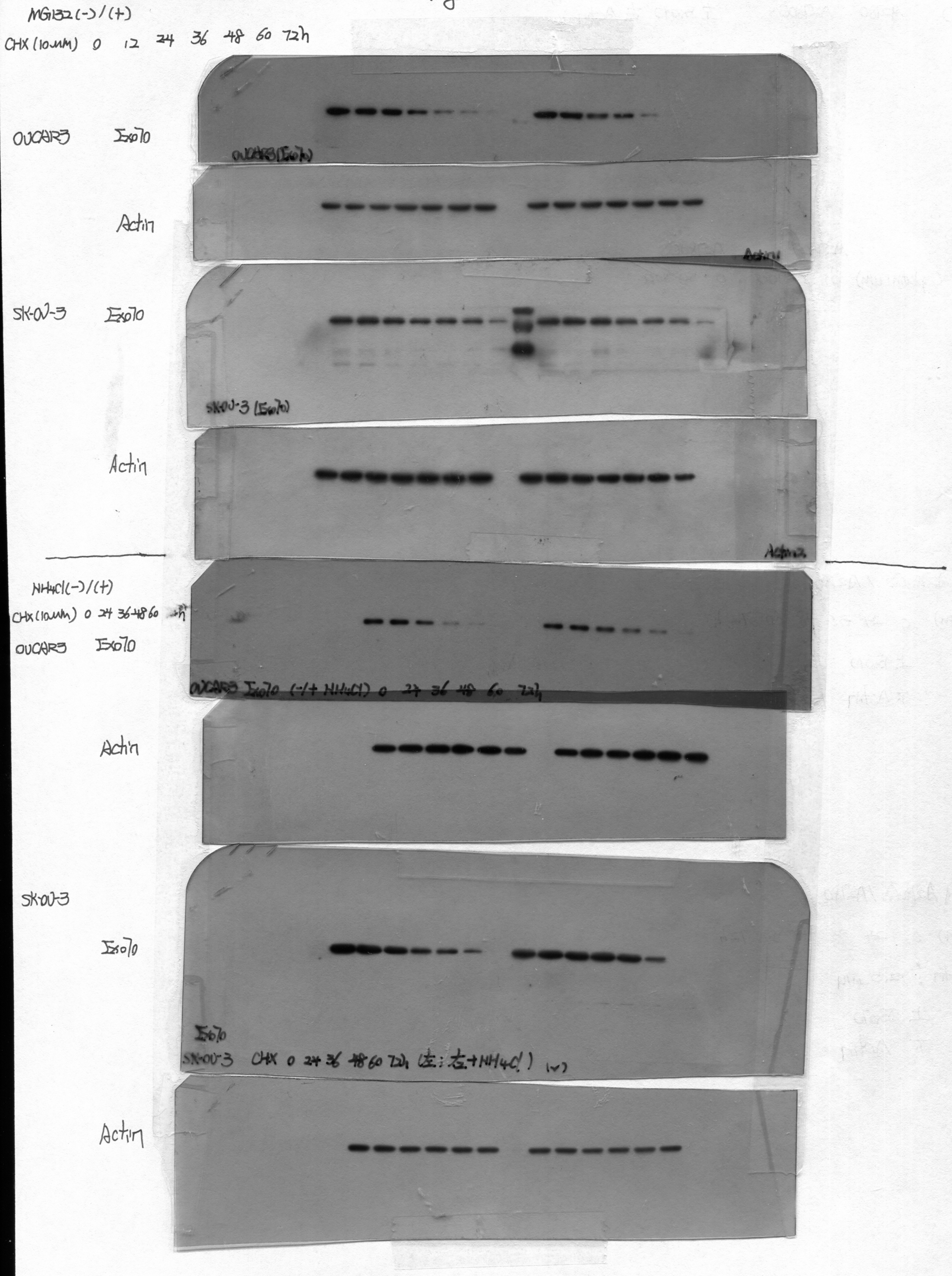
MG132(-)/(+)
CHX (10uM) 0 12 24 36 48 60 72h
OVCAR3 Exo70
OVCAR3 (Exo70)
Actin
SK-OV-3 Exo70
SK-OV-3 (Exo70)
Actin
NH4Cl(-)/(+)
CHX (10uM) 0 24 36 48 60 72h
OVCAR3 Exo70
OVCAR3 Exo70 (-/+ NH4Cl) 0 24 36 48 60 72h
Actin
SK-OV-3
Exo70
Exo70
SK-OV-3 CHX 0 24 36 48 60 72h (左: 左+NH4Cl)
Actin

Fig S5

A2780

MG132 (-)/(+) Cisplatin (25 uM) 上 Exo70

CHX (10 uM) 0 12 24 36 48 72 h 下 Actin.

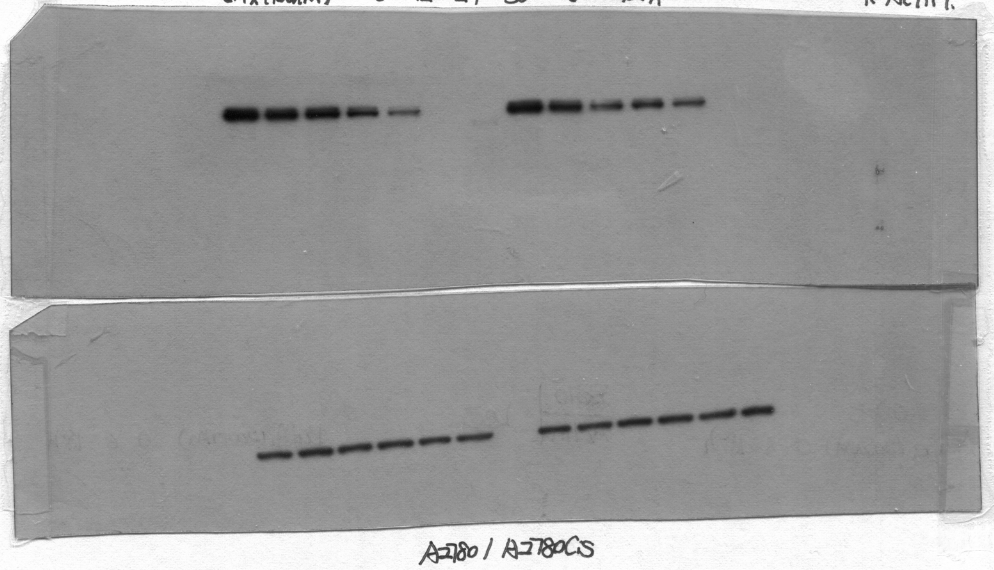

A2780 / A2780CS

左→右 Exo70 Beclin1 BCl2 β-actin.

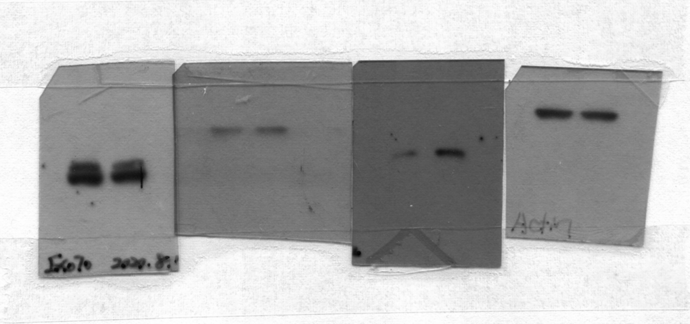


---

Figure 5

①

A2780

MG132 (-)/(+)

CHX 0 12 24 36 48 60 72 h.

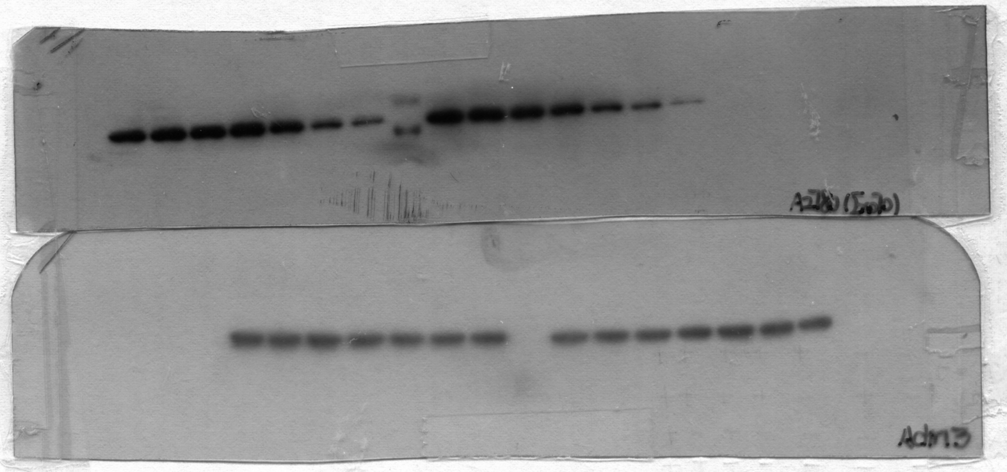


②

A2780

NH4Cl (-)/(+)

CHX 0 24 36 48 60 72 h.

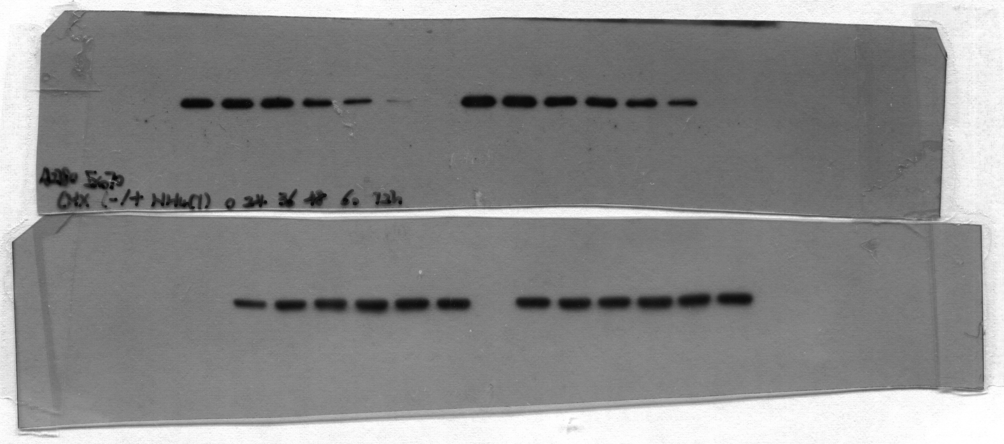

③ A2780/A2780Cis    Exo70 | LC3
Actin

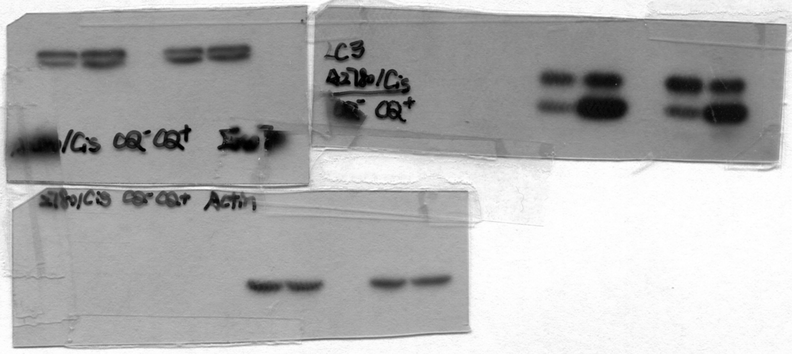


SKOV-3
Rapa (20uM) 0 6 18 h    Exo70 / Actin | LC3

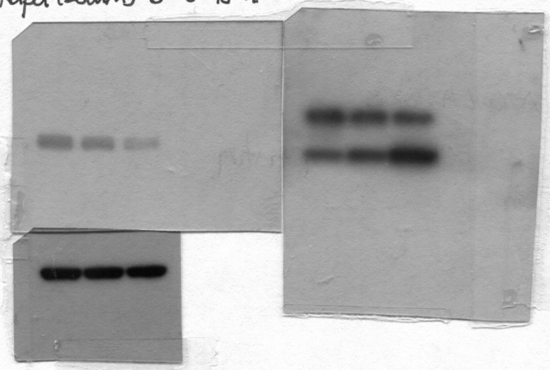

BafA1 (200nM) 0 6 18h    Exo70 | LC3 / Actin

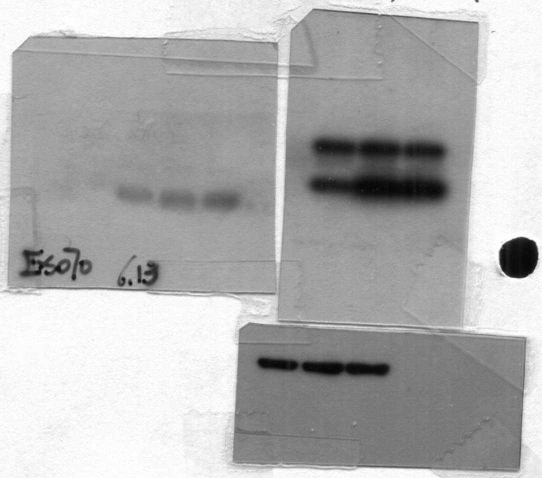


④ A2780
shCtrl shATG5-1 shATG5-2
上ATG5 下Actin

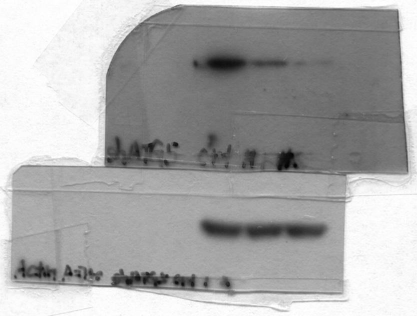

⑤ A2780
shCtrl / shATG5-1 / shATG5-2
CHX (10uM) 0 24 48 72h

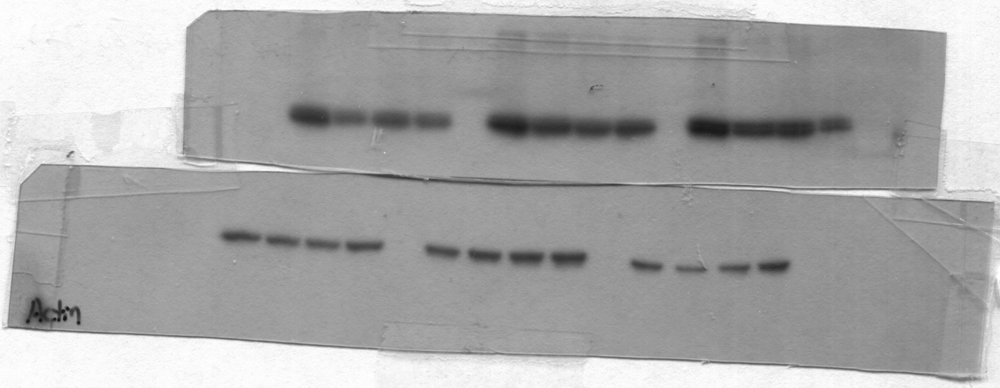


⑥

A2780

| | | | | | | | |
|---|---|---|---|---|---|---|---|
| Ub-Myc | - | - | + | + | + | + | + |
| Exo70-Flag | - | + | - | + | + | + | + |
| CQ | - | - | - | - | + | - | + |
| MG132 | - | - | - | - | - | + | + |

IP
Flag-Exo70

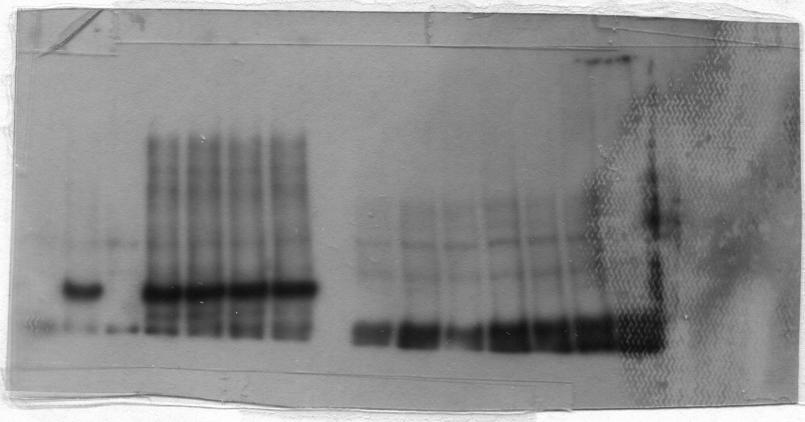

Co-IP
Myc-Ub

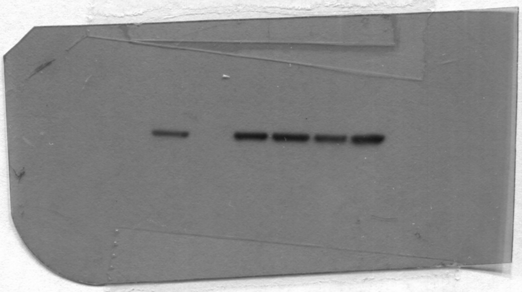

Flag
(Input)

Figure 5

⑦ A2780 Cisplatin 25uM

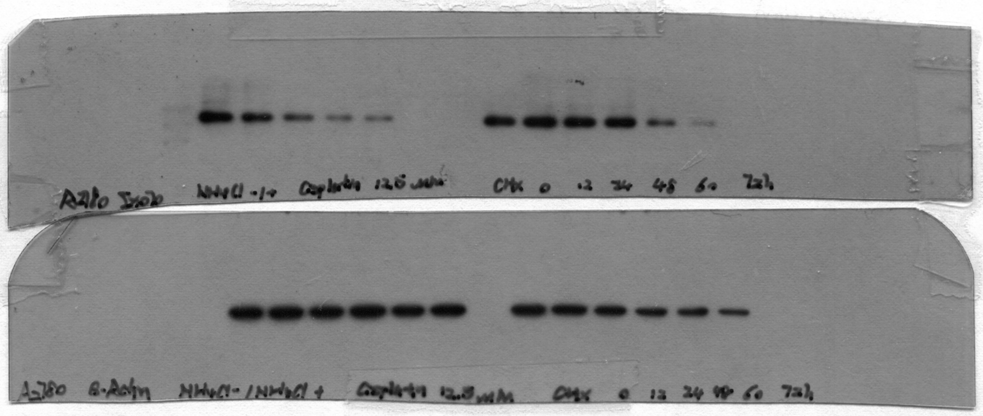

⑧ A2780 Cisplatin 50uM

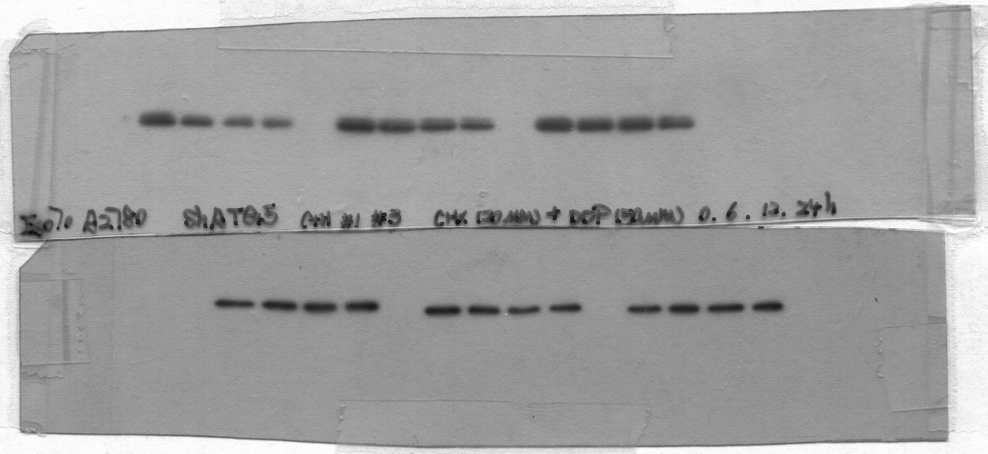

⑨ A2780 / A2780Cis

CQ⁻ CQ⁺

Cisplatin (uM) 0 25 50 100

左 A2780 右 A2780Cis

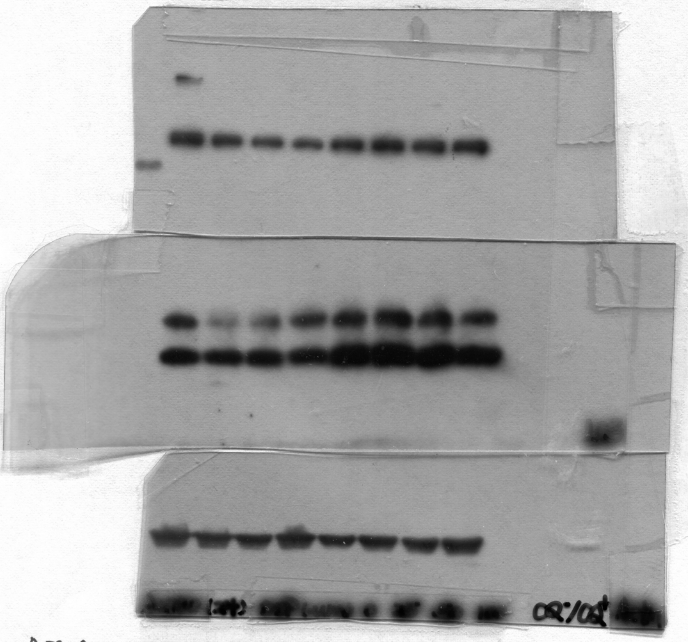

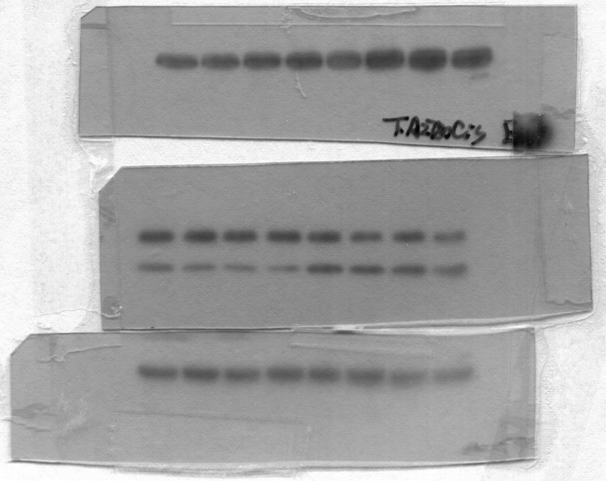

⑩ A2780Cis

Cisplatin + −

Rap − + − +

CQ + + + +

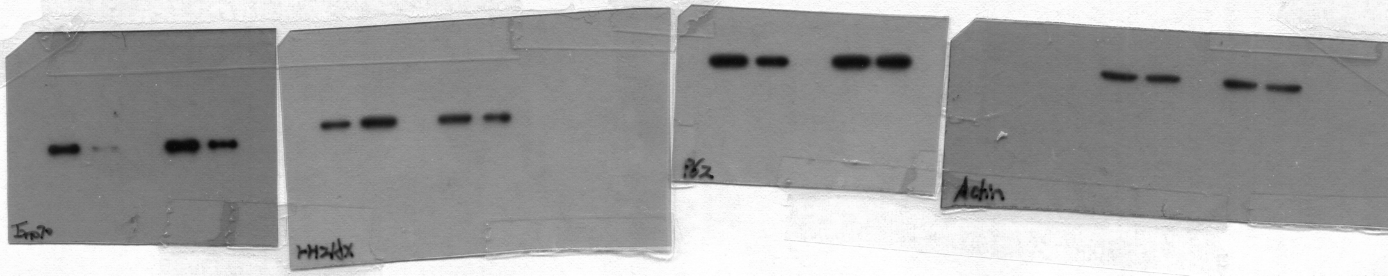

(11)

A2780 A2780Cis

Cisplatin (uM) 0 25 50 0 25 50

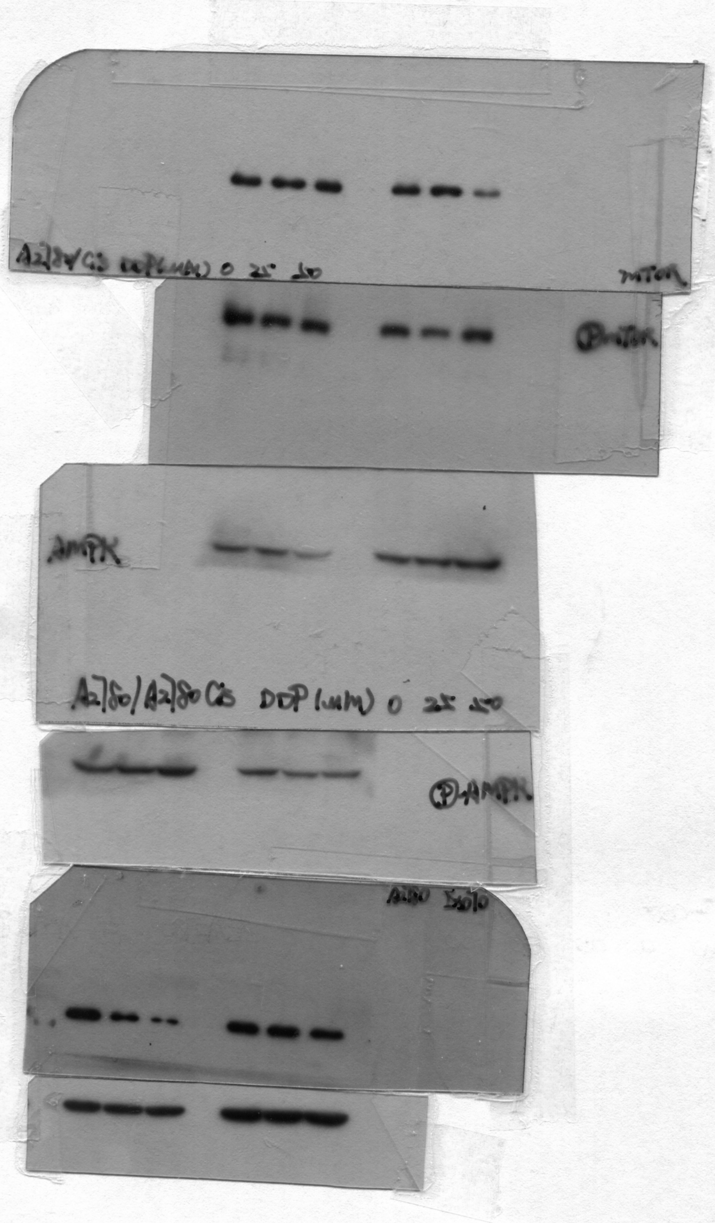

# Figure 6

① A2780Cis shCtrl shExo70-1 shExo70-2

Exo70

Actin

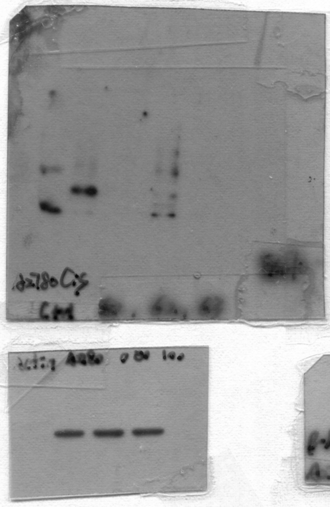

② shCtrl shExo70-1 shExo70-2

Cisplatin (uM) 0 25 50 100

上 rH2AX

下 Actin.

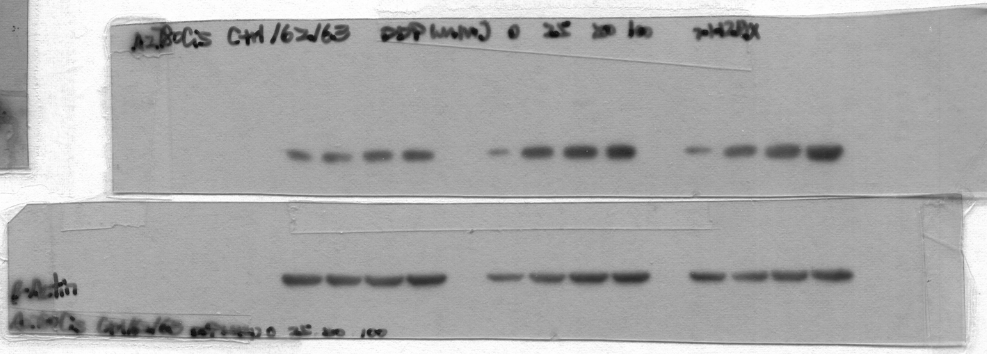

③ A2780Cis

shCtrl shExo70-1 shExo70-2.

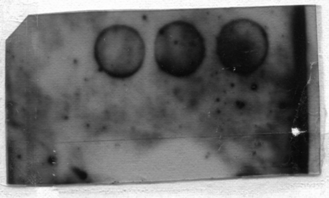

④ A2780Cis

ES (-) / (+)

Cisplatin - + - +

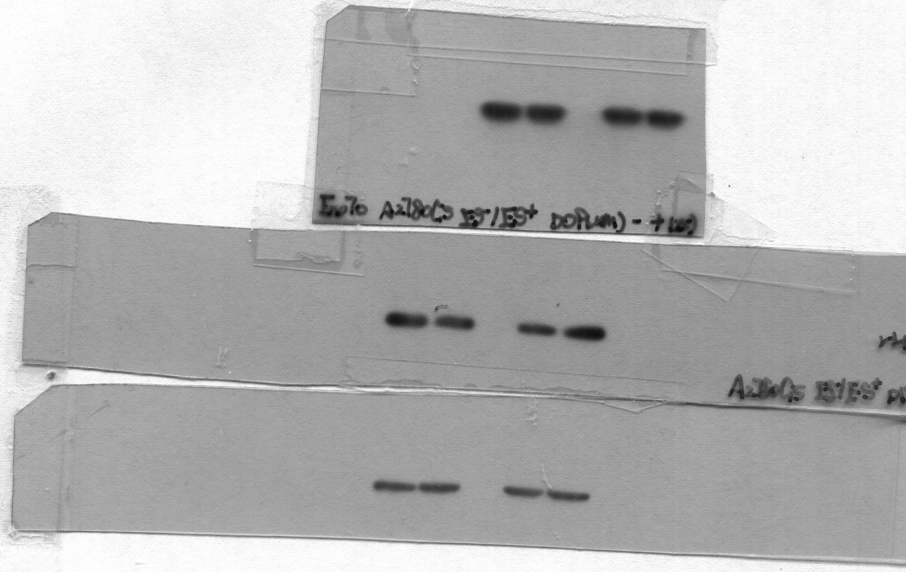

Supplement: Supplementary file 1 [file cancers-13-03467-s001.zip › Figure S7 Original Images for Blots Gels .pdf]
