# Supplementary material for: Deregulation of Exo70 Facilitates Innate and Acquired Cisplatin Resistance in Epithelial Ovarian Cancer by Promoting Cisplatin Efflux
Source: Cancers (Basel). 2021 Jul 11;13(14):3467. doi: 10.3390/cancers13143467 (PMC8304026; doi:10.3390/cancers13143467)
Supplement: Supplementary file 1 [file cancers-13-03467-s001.zip › Figure S8 STR profile for cell lines-done.pdf]

cancers

Supplementary Materials

Deregulation of Exo70 Facilitates Innate and Acquired Cisplatin Resistance in Epithelial Ovarian Cancer by Promoting Cisplatin Efflux

	Test Results for Submitted Sample			DSMZ Reference Database Profile	
Loci	Query Profile: S			Database Profile: SK-OV-3	
Amelogenin	X			X	
D3S1358	14				
D13S317	8	11		8	11
D7S820	13	14		13	14
D16S539	12			12	
Penta E	5	13			
TPOX	8	11		8	11
TH01	9	9.3		9	9.3
D2S1338	18	23			
CSF1PO	11			11	
Penta D	12	13			
D19S433	14	14.2			
vWA	17	18		17	18
D21S11	30	31.2			
D18S51	16	17	18		
D6S1043	12				
D8S1179	14	15			
D5S818	11			11	
D12S391	22				
FGA	24	25			

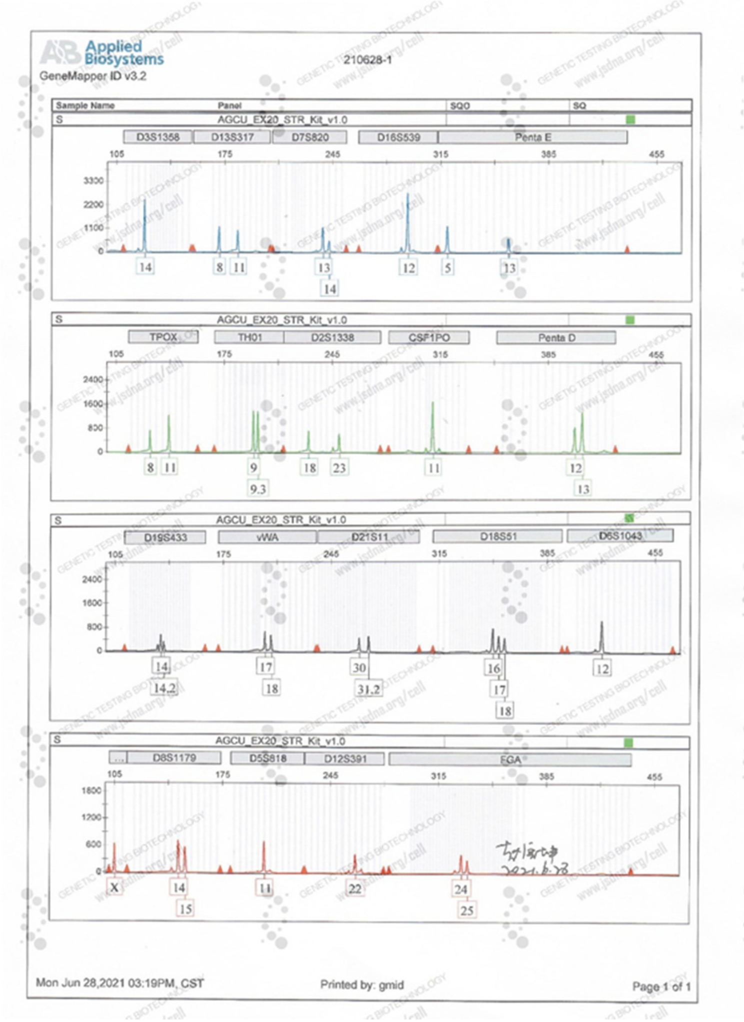

	Test Results for Submitted Sample		DSMZ Reference Database Profile	
Loci	Query Profile: 3-166		Database Profile: NIH:OVCAR-3	
Amelogenin	X		X	
D3S1358	17	18		
D13S317	12		12	
D7S820	10		10	
D16S539	12		12	
Penta E	7	13		
TPOX	8		8	
TH01	9	9.3	9	9.3
D2S1338	17	21		
CSF1PO	11	12	11	12
Penta D	12	13		
D19S433	16.2			
vWA	17		17	
D21S11	29	31.2		
D18S51	13			
D6S1043	10	11		
D8S1179	10	15		
D5S818	11	12	11	12
D12S391	19	22		
FGA	21			

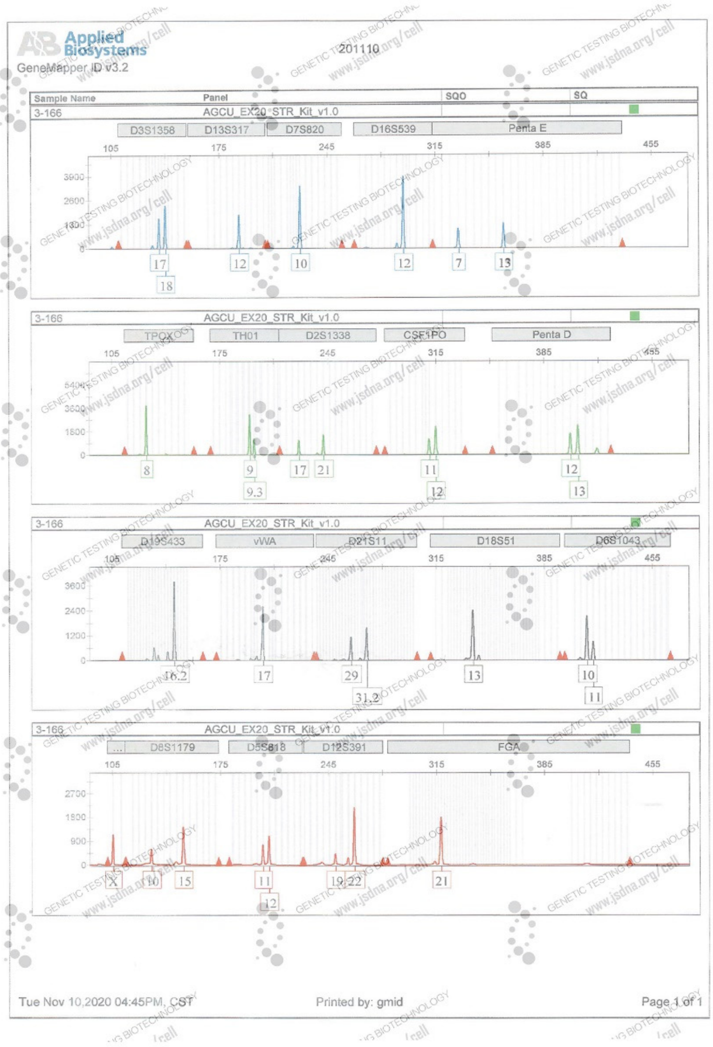

	Test Results for Submitted Sample		ExPASy Reference Database Profile	
Loci	Query Profile: 3-11		Database Profile: A2780	
Amelogenin	X		X	
D3S1358	14	16		
D13S317	12	13	12	13
D7S820	10		10	
D16S539	11	13	11	13
Penta E	10	13		
TPOX	8	10	8	10
TH01	6		6	
D2S1338	21	22		
CSF1PO	10	11	10	11
Penta D	8	9		
D19S433	12			
vWA	15	16	15	16
D21S11	28	29		
D18S51	17			
D6S1043	11	17		
D8S1179	15	17		
D5S818	*10*	*13*	*11*	*12*
D12S391	19	20		
FGA	19	24		

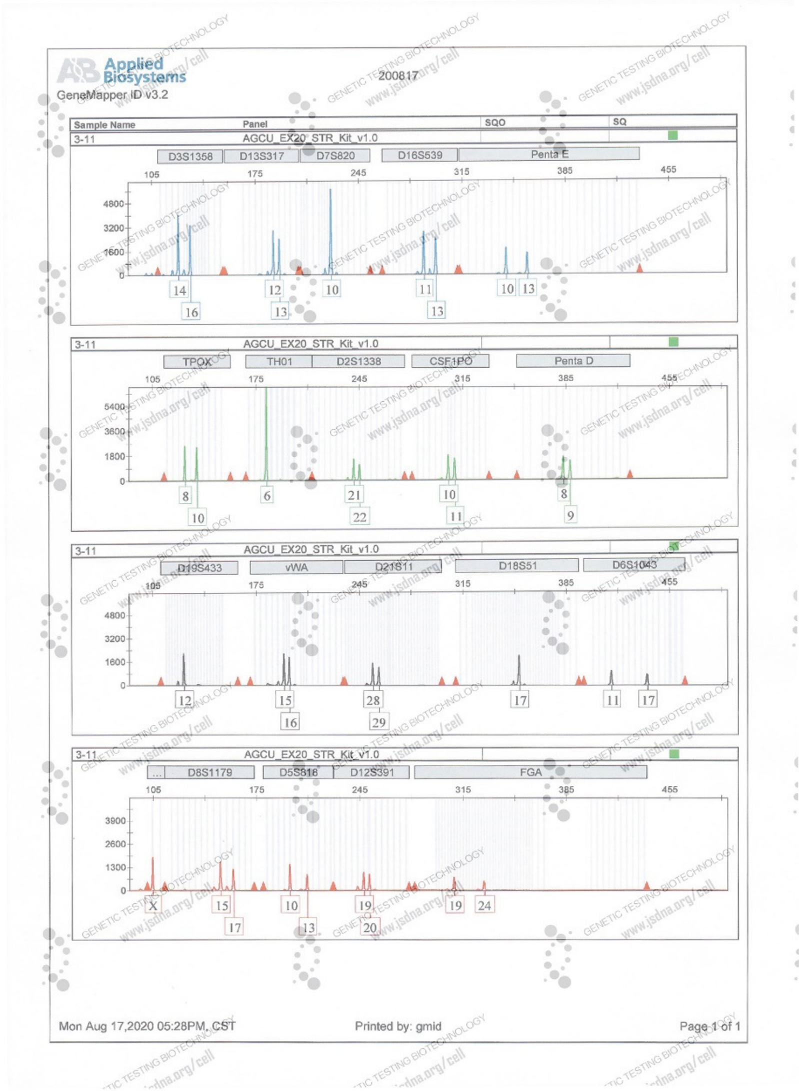

Figure S8. STR profile for cell lines.
