# Supplementary material for: Deregulation of Exo70 Facilitates Innate and Acquired Cisplatin Resistance in Epithelial Ovarian Cancer by Promoting Cisplatin Efflux
Source: Cancers (Basel). 2021 Jul 11;13(14):3467. doi: 10.3390/cancers13143467 (PMC8304026; doi:10.3390/cancers13143467)
Supplement: Supplementary file 1 [file cancers-13-03467-s001.zip › Supplementary Figures S1-6+Supplementary Tables-done.pdf]

cancers

MDPI

Supplementary Materials:

Deregulation of Exo70 Facilitates Innate and Acquired Cisplatin Resistance in Epithelial Ovarian Cancer by Promoting Cisplatin Efflux

Yujie Zhao, Xiaoting Hong, Xiong Chen, Chun Hu, Weihong Lu, Baoying Xie, Linhai Zhong, Wenqing Zhang, Hanwei Cao, Binbin Chen, Qian Liu, Yanyan Zhan, Li Xiao and Tianhui Hu

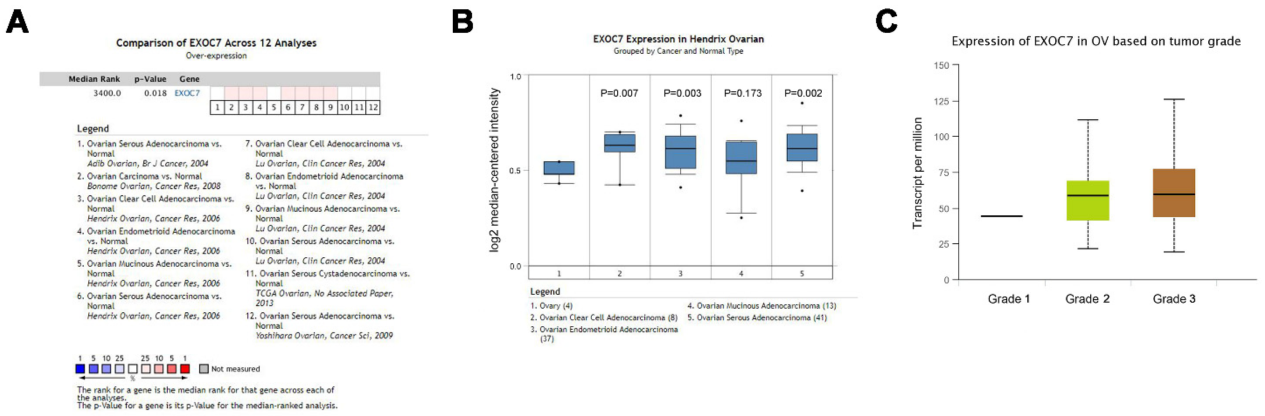

Figure S1. EXOC7 was highly expressed in EOC samples. (**A**) Comparison of EXOC7 between ovarian cancer and normal tissue across 12 analyses by Oncomine database. (**B**) EXOC7 was highly expressed in EOC samples compare to normal ovary tissue. Data were generated from the Hendrix Ovarian dataset (Serous vs normal *P* = 0.007; Mucinous vs normal *P* = 0.003; Endometrioid vs. normal *P* = 0.173; Clear cell vs. normal *P* = 0.002). (**C**) The expression level of EXOC7 increased with the EOC progressions. Data were generated from the UALCAN database.

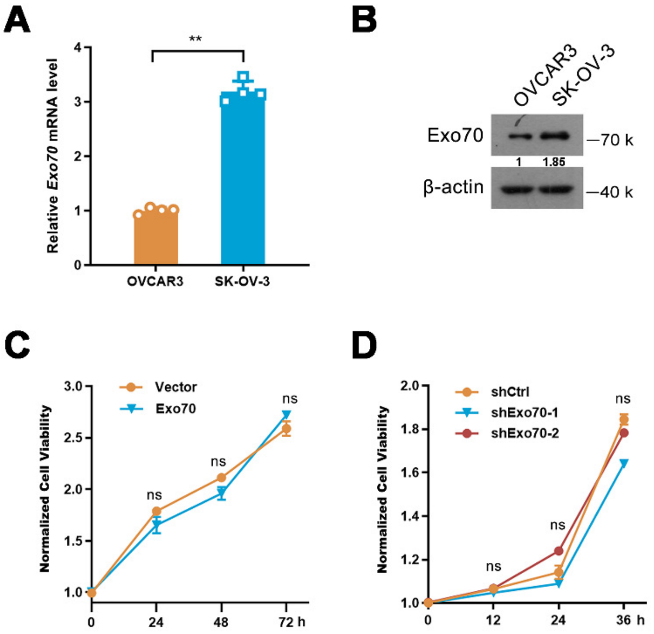

Figure S2. Exo70 expression did not affect the growth ratio of EOC cells. (**A**) RT-PCR showed the Exo70 mRNA levels in OVCAR3 and SK-OV-3 cells. Data were expressed as mean ± SEM, *n* = 3. **, *P* < 0.01. (**B**) Western blotting showed the Exo70 protein expressions in OVCAR3 and SK-OV-3 cells. (**C**) CCK8 assay measured the effect of overexpressing Exo70 on the proliferation of OVCAR3 cells; Data were expressed as mean ± SEM, *n* = 3. ns: not significant. (**D**) CCK8 assay was carried out to measure the effect of knockdown Exo70 on the proliferation of SK-OV-3 cells. Data were expressed as mean ± SEM, *n* = 3. ns: not significant.

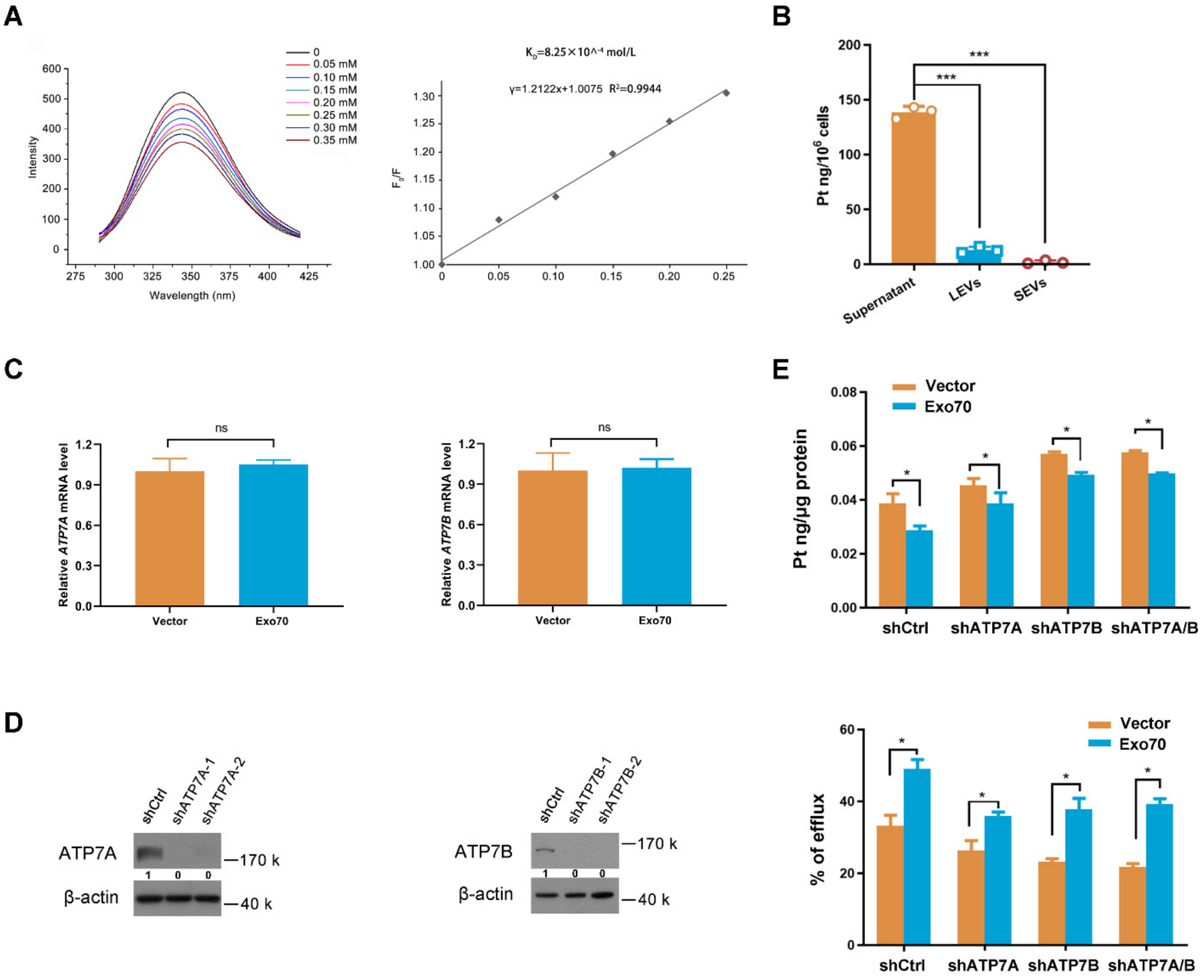

Figure S3. Exo70 efflux cisplatin to supernatant independent of ATP7A and ATP7B. (A) Fluorescence quenching assay was carried out to exam the binding ability of Exo70 protein and cisplatin. KD = 8.25 × 10^{-4} mol/L. (**B**) ICP-MS assay was carried out to test the cisplatin distributions in different components of the culture medium. Data were expressed as mean ± SEM, *n* = 3. ***, *P* < 0.001. LEVs: large extracellular vesicles, SEVs: small extracellular vesicles. (**C**) RT-PCR showed the ATP7A and ATP7B mRNA levels in cells. Data were expressed as mean ± SEM, *n* = 3. ns: not significant. (**D**) The knockdown of ATP7A or ATP7B by shRNA in OVCAR3 cells were shown by western blotting. (**E**) ICP-MS assays were carried out to test the effects of knockdown ATP7A or ATP7B on the intracellular and extracellular cisplatin distributions in OVCAR3 cells. Data were expressed as mean ± SEM, *n* = 3. *, *P* < 0.05.

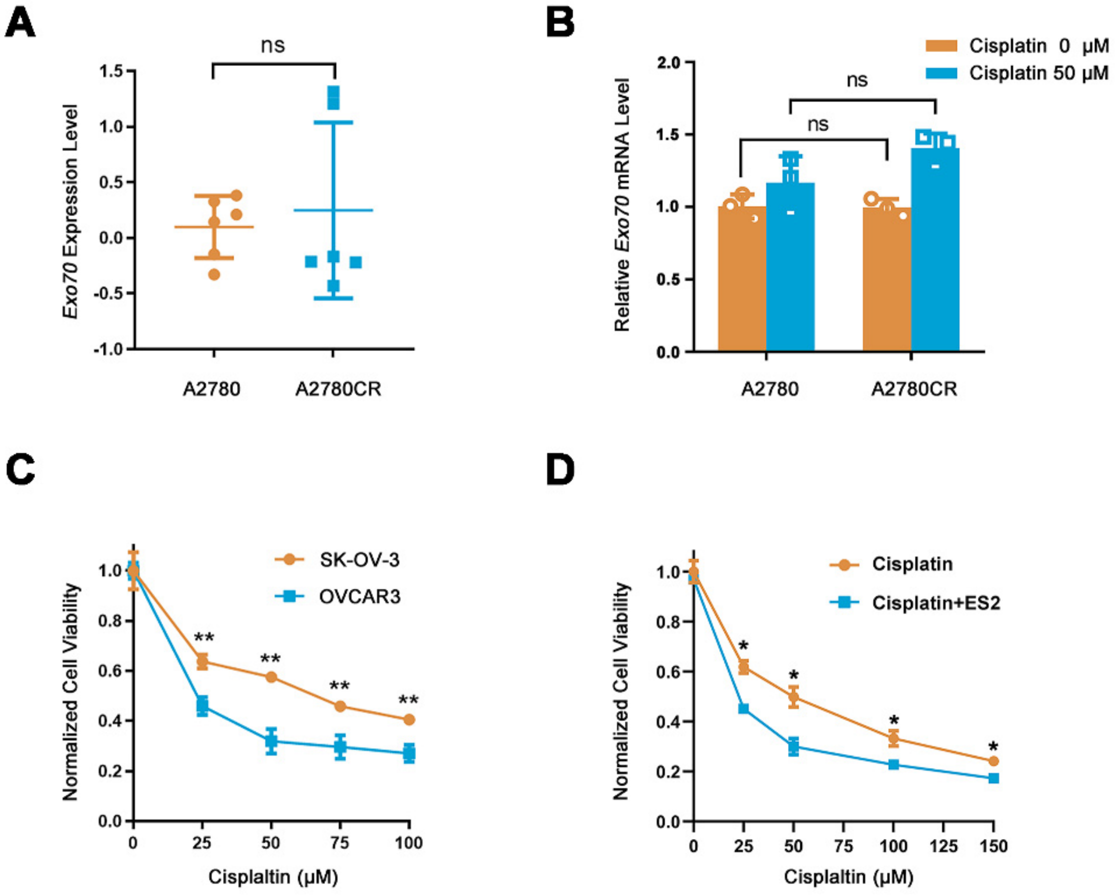

Figure S4. Exo70 affected the sensitivity of cells to cisplatin. (**A**) Exo70 mRNA expression levels were evaluated in datasets from GEO (GSE 33482) by comparing cisplatin-sensitive A2780 cells vs cisplatin-resistant A2780 cells. (**B**) RT-PCR showed the Exo70 mRNA expression levels in A2780 and A2780CR cells. Data were expressed as mean ± SEM, $n = 3$. ns: not significant. (**C**) CCK8 assay showed the cytotoxicity of cisplatin in OVCAR3 and SK-OV-3 cells. Cells were treated with different doses of cisplatin for 24 hours. **, $P < 0.01$. (**D**) CCK8 assay was carried out to examine the cytotoxicity of cisplatin in SK-OV-3 cells with or without ES2 (100 μM) treatment. Data were presented as mean ± SEM, $n = 4$. **, $P < 0.01$.

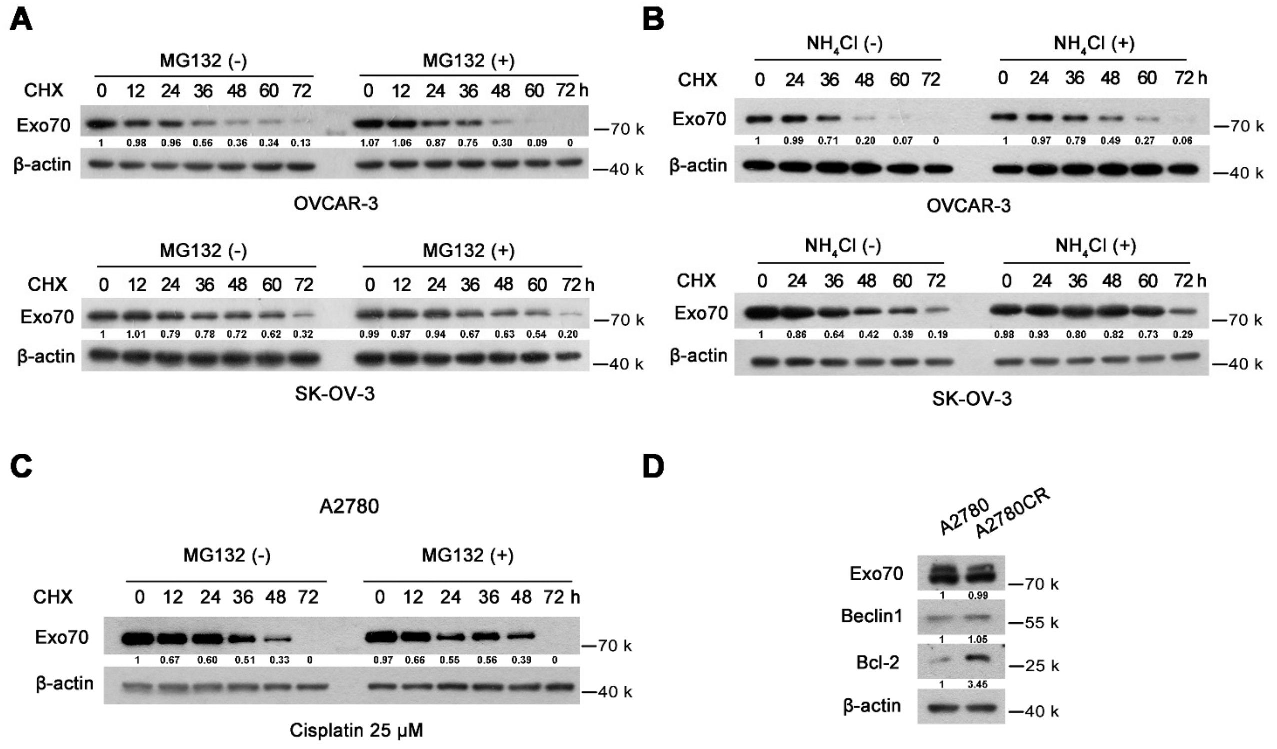

Figure S5. Autophagy affected the protein stability of Exo70. (**A**) Proteasome inhibitor MG132 did not alter the protein stability of Exo70 in OVCAR3 and SK-OV-3 cells. Cells were treated with 10 μM CHX together with or without 20 μM MG132. One of three experiments was shown. (**B**) Lysosome inhibitor NH4Cl altered the protein stability of Exo70 in OVCAR3 and SK-OV-3 cells. Cells were treated with 10 μM CHX together with or without 10 mM NH4Cl. One of three experiments was shown. (**C**) Western blotting was used to detect the half-lives of Exo70 in A2780 cells with 25 μM cisplatin treatment together with or without MG132. (**D**) Western blotting showed the expression level of Beclin1 and Bcl-2 in A2780 and A2780CR cells. One of three experiments was shown.

Table S1. Information of Antibodies.

Antibody	Company	Product Number	RRID
Anti-Exo70 (Rabbit)	Abcam	118792	RRID:AB_10901372
Anti-Exo70 (Goat)	Abcam	77352	RRID:AB_1523581
IgG-Isotype (Goat)	Abcam	172730	RRID:AB_2687931
Anti-LC3B (Rabbit)	Novus	NB100-2220	RRID:AB_10003146
Anti-phospho-Histone H2A.X (Rabbit)	CST	9720	RRID:AB_10692910
Anti-mTOR (Rabbit)	CST	2972	RRID:AB_330978
anti-phospho-mTOR (Ser2448) (Rabbit)	CST	5536	RRID:AB_10691552
anti-AMPKα (Rabbit)	CST	2532	RRID:AB_330331
anti-phospho-AMPKα (Thr172) (Rabbit)	CST	2535	RRID:AB_331250
Anti-Ubiquitin (Rabbit)	CST	3933	RRID:AB_2180538
Anti-ATG5 (Rabbit)	CST	12994	RRID:AB_2630393
Anti-ATG7 (Rabbit)	CST	8558	RRID:AB_10831194
Anti-ATG12 (Rabbit)	CST	4180	RRID:AB_1903898
Anti-ATG16L1 (Rabbit)	CST	8089	RRID:AB_10950320
Anti-Beclin1 (Rabbit)	CST	3495	RRID:AB_1903911
Anti-BCl2 (Mouse)	CST	15071	RRID:AB_2744528
Anti-PIK3R4 (Rabbit)	CST	14580	RRID:AB_2798523
Anti-ATP7A (Mouse)	Santa Cruz Biotechnology	sc-376467	RRID:AB_11150485

Anti-ATP7B (Mouse)	Santa Cruz Biotechnology	sc-373964	RRID:AB_10920224
Anti-Sec3 (Rabbit)	Proteintech	11690-1-AP	RRID:AB_2231500
Anti-Sec5 (Rabbit)	Proteintech	12723-1-AP	RRID:AB_10734442
Anti-Sec15 (Rabbit)	Proteintech	18420-1-AP	RRID:AB_10694431
Anti-Flag (Mouse)	Sigma-Aldrich	F3165	RRID:AB_259529
Anti-HA (Mouse)	Sigma-Aldrich	H9658	RRID:AB_260092
Anti-c-Myc (Mouse)	Sigma-Aldrich	M4439	RRID:AB_439694
Anti-Cisplatin DNA adducts (Rabbit)	Sigma-Aldrich	MABE416	

Table S2. Sequences of oligonucleotide primers for shRNA construction.

Target	Sequence
shExo70#1	5′-GCACAGAGACCGTGGGCAA-3′
shExo70#2	5′-GCTGGTGTTGTACATGAA-3′
shATG5#1	5′-ATATCAGGATGAGATAAC-3′
shATG5#2	5′-GCAGTGGCTGAGTGAACATCT-3′
shATG7#1	5′-GCTGCCTGTCCTGAAATAGAT-3′
shATG7#2	5′-GCAGAACAATTCCTTGCATTC-3′
shSec3#1	5′-TTTGGAGTTCTTCAGCTT-3′
shSec3#2	5′- GAGTGGCTAAAGAGTACAGAT-3′
shSec5#1	5′-GCCTGGTATCTTATAGAGAAT-3′
shSec5#2	5′- CAATGTGCTTCAGCGATTTAA-3′
shSec15#1	5′- GCAGTTTAACTTAGATGTCAT-3′
shSec15#2	5′- CCCTTTCAAGATCCAGACCTT-3′
shATP7A#1	5′- GGAAGGGATGACCTGCCATTC-3′
shATP7A#2	5′- GCAGCCCAAGTACCTCAAATT-3′
shATP7B#1	5′-GCAGTGCCACTGTGAAATATG-3′
shATP7B#2	5′-GGGACCATGTAAATGACATGG-3′

Table S3. Sequences of primers for real-time PCR.

Gene	Primers
GAPDH	Forward：5′-TGCACCACCAACTGCTTAGC -3′
	Reverse：5′-GGCATGGACTGTGGTCATGAG -3′
EXOC7	Forward：5′-GGAGTATTTCCAGGACAACAGC -3′
	Reverse：5′- AAGATGAGCACGGGCGAGA -3′
ATG5	Forward：5′- AACTGAAAGGGAAGCAGAACCA -3′
	Reverse：5′- ATGCCATTTCAGTGGTGTGCC -3
ATG7	Forward：5′-GCTTGTGCCTCACCAGGTTC -3′
	Reverse：5′- GATGGTCTCATCATCGCTCATGTC -3′
EXOC1	Forward：5′-ACCAAATGATGAACGCCTGC -3′
	Reverse：5′- GGCATCTACCACAGCAAGAT -3′
EXOC2	Forward：5′-AAGAACTGGTCCCTCGGGTA -3′
	Reverse：5′- TCGGGTGTCAGGTAAACAGC -3′
EXOC6	Forward：5′-CGGGTGAATCCAAACACAGC -3′
	Reverse：5′- TCAGCTGTTTCACGACTGTCT -3′
ATP7A	Forward：5′-TGGACCATTGAGCAGCAGAT -3′
	Reverse：5′- GTGACGCCGTAACAGTCAGA -3′
ATP7B	Forward：5′-TGGTCACCCTCCAACTGAGA -3′
	Reverse：5′- GGGCTGGTACAAGAAGGGTC -3′

Table S4. Characteristics of the ten ovarian cancer patients.

	**Platinum-sensitive**	
**Serial Number**	**Stage**	**Pathological Type**
1	II c	Serous ovarian cancer
2	III b	Serous ovarian cancer
3	III c	Serous ovarian cancer
4	III c	Serous ovarian cancer
5	III c	Serous ovarian cancer
	**Platinum-resistant**	
**Serial Number**	**Stage**	**Pathological Type**
1	III a	Serous ovarian cancer
2	III c	Serous ovarian cancer
3	III c	Serous ovarian cancer
4	III c	Serous ovarian cancer
5	IV	Serous ovarian cancer

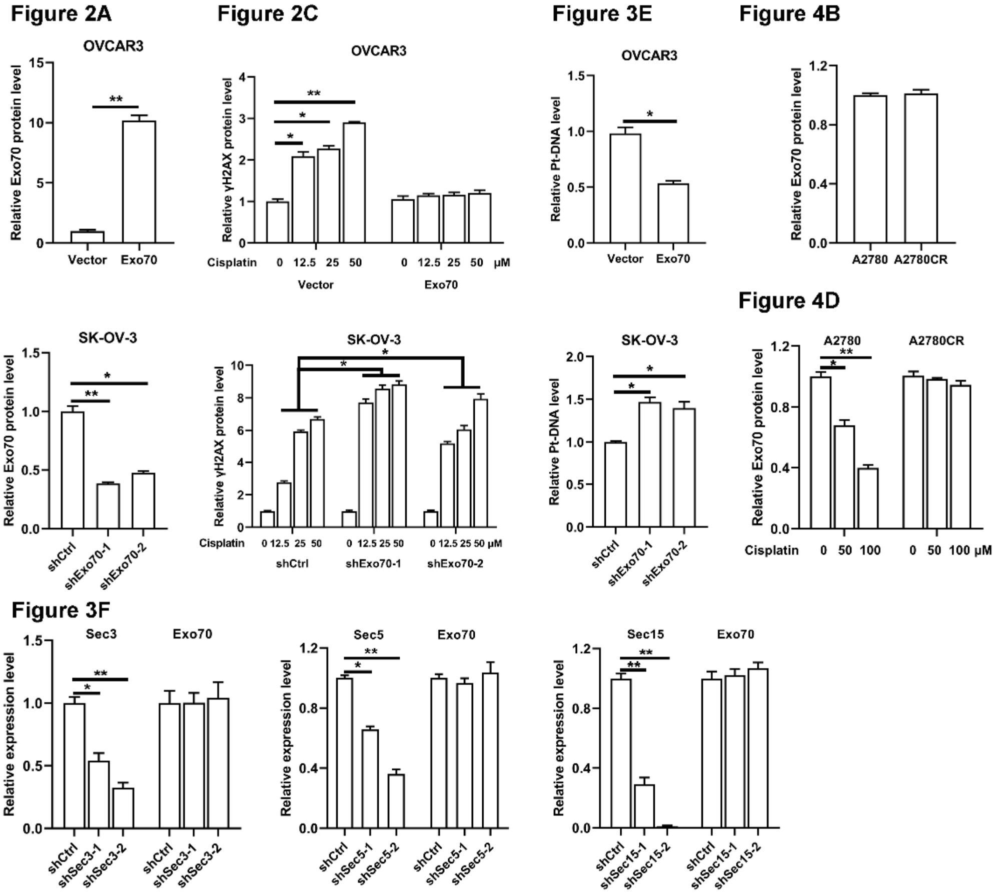

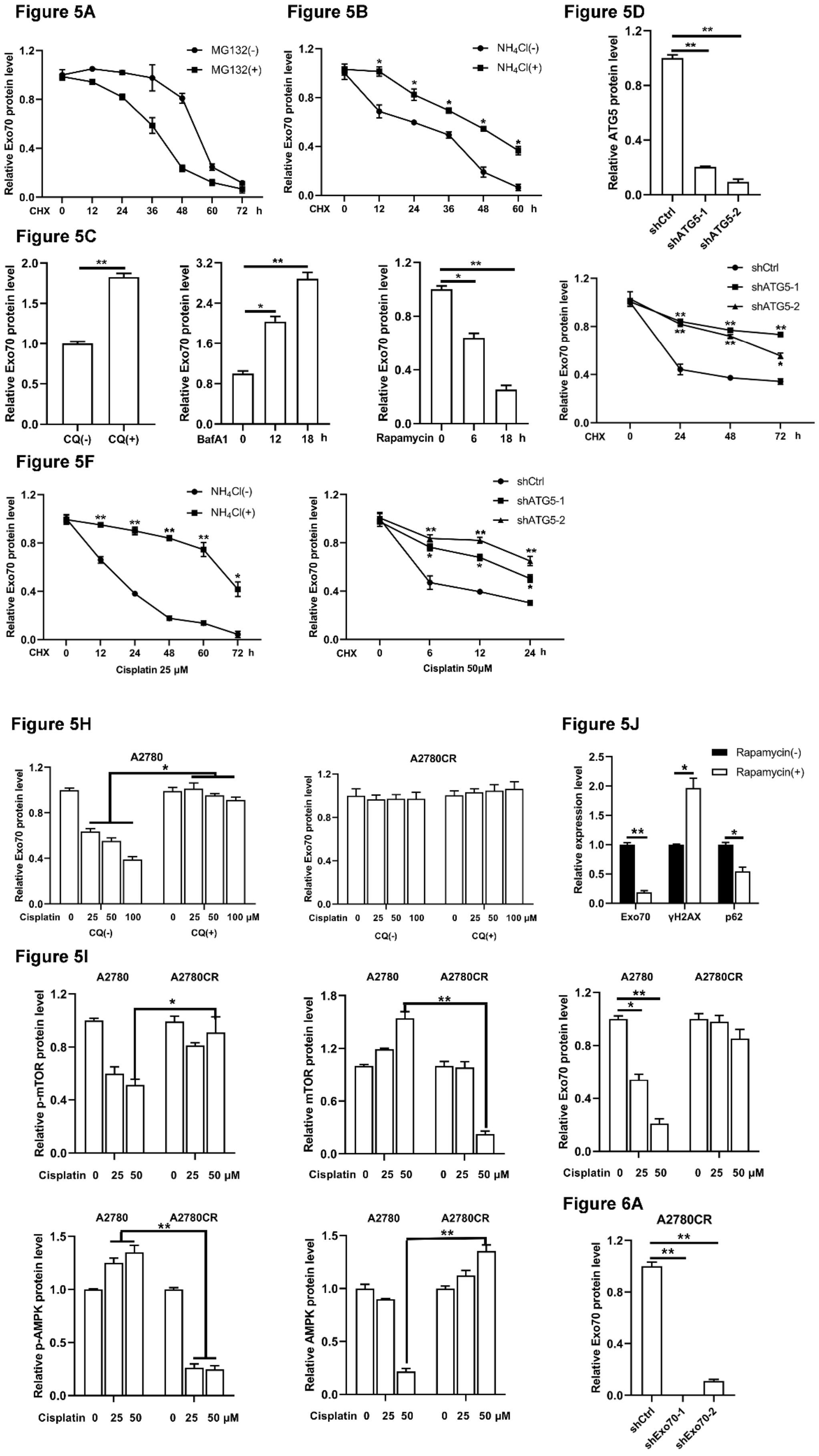
Figure 5A
Relative Exo70 protein level
MG132(-)
MG132(+)
CHX 0 12 24 36 48 60 72 h
Figure 5B
NH4Cl(-)
NH4Cl(+)
CHX 0 12 24 36 48 60 h
Figure 5D
Relative ATG5 protein level
shCtrl
shATG5-1
shATG5-2
Figure 5C
CQ(-) CQ(+)
BafA1 0 12 18 h
Rapamycin 0 6 18 h
CHX 0 24 48 72 h
Figure 5F
CHX 0 12 24 48 60 72 h
Cisplatin 25 μM
CHX 0 6 12 24 h
Cisplatin 50μM
Figure 5H
A2780
A2780CR
Cisplatin 0 25 50 100 0 25 50 100 μM
CQ(-) CQ(+)
Figure 5J
Relative expression level
Rapamycin(-)
Rapamycin(+)
Exo70 γH2AX p62
Figure 5I
Relative p-mTOR protein level
Relative mTOR protein level
Relative p-AMPK protein level
Relative AMPK protein level
Cisplatin 0 25 50 0 25 50 μM
Figure 6A
A2780CR
shCtrl
shExo70-1
shExo70-2

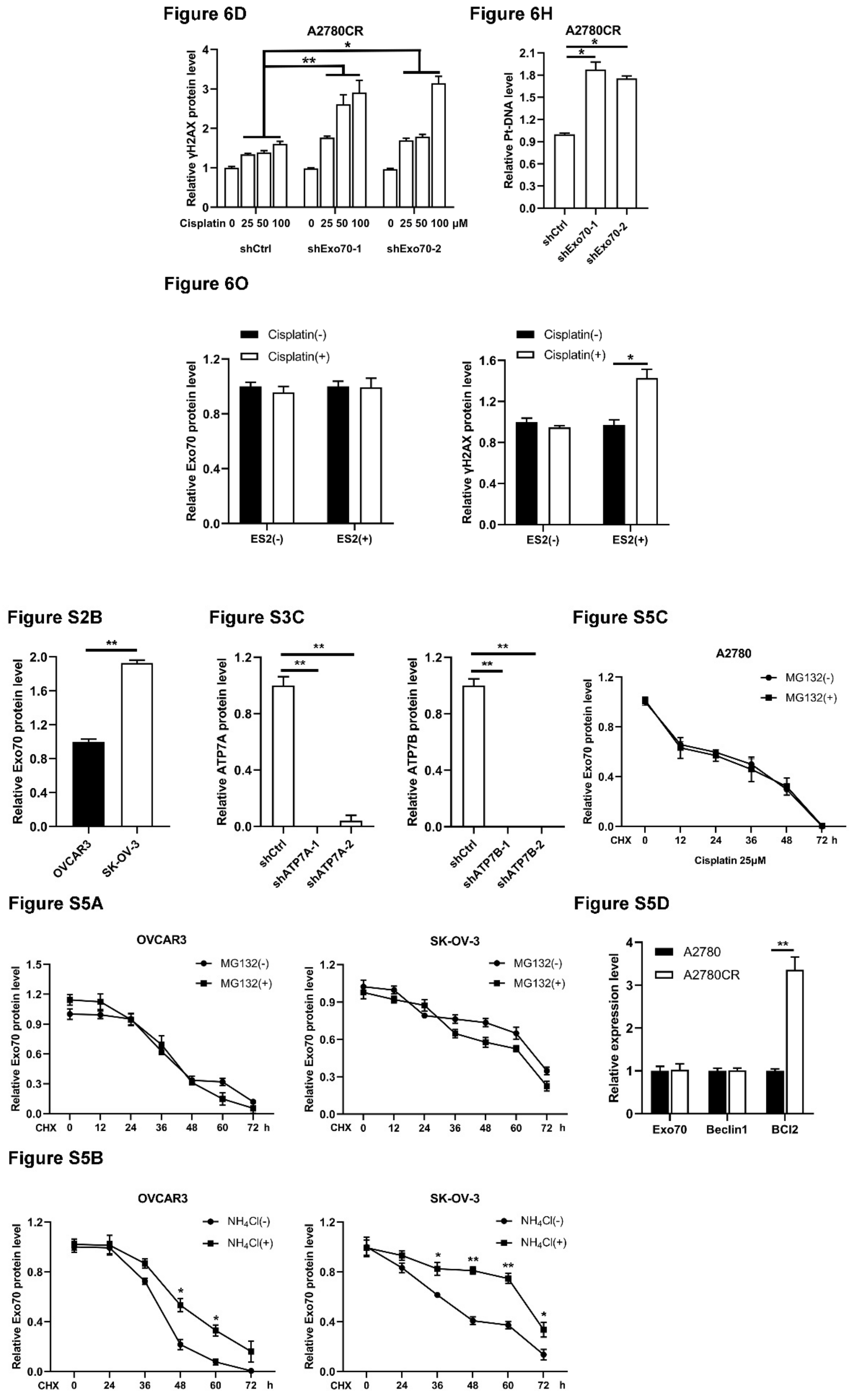

Figure S6. Quantification of Western Blot. Data were expressed as mean ± SEM, *n* = 3. * P < 0.05, ** P < 0.01.
